# Challenges and opportunity in mobility among older adults – key determinant identification

**DOI:** 10.1186/s12877-023-04106-7

**Published:** 2023-07-21

**Authors:** Petra Maresova, Ondrej Krejcar, Raihan Maskuriy, Nor Azlina Abu Bakar, Ali Selamat, Zuzana Truhlarova, Jiri Horak, Miroslav Joukl, Lucie Vítkova

**Affiliations:** 1 Faculty of Informatics and Management, University of Hradec Kralove, Rokitanskeho 62, Hradec Kralove, 500 03 Czech Republic; 2 Malaysia-Japan International Institute of Technology, Universiti Teknologi Malaysia Kuala Lumpur, Jalan Sultan Yahya Petra, 54100 Kuala Lumpur, Malaysia; 3 Universiti Putra Malaysia, 54100 Kuala Lumpur, Malaysia; 4 Faculty of Education, University of Hradec Kralove, Rokitanskeho 62, Hradec Kralove, 500 03 Czech Republic; 5 Faculty of Mining and Geology, VSB—Technical University of Ostrava, 17. Listopadu 2172/15, Ostrava-Poruba, 708 00 Czech Republic; 6 Philosophical Faculty, University of Hradec Kralove, Rokitanskeho 62, Hradec Kralove, 500 03 Czech Republic

**Keywords:** Mobility, Determinants, Older adults, Financial aspects, Technological solutions

## Abstract

**Background:**

Attention is focused on the health and physical fitness of older adults due to their increasing age. Maintaining physical abilities, including safe walking and movement, significantly contributes to the perception of health in old age. One of the early signs of declining fitness in older adults is limited mobility. Approximately one third of 70-year-olds and most 80-year-olds report restrictions on mobility in their apartments and immediate surroundings. Restriction or loss of mobility is a complex multifactorial process, which makes older adults prone to falls, injuries, and hospitalizations and worsens their quality of life while increasing overall mortality.

**Objective:**

The objective of the study is to identify the factors that have had a significant impact on mobility in recent years and currently, and to identify gaps in our understanding of these factors. The study aims to highlight areas where further research is needed and where new and effective solutions are required.

**Methods:**

The PRISMA methodology was used to conduct a scoping review in the Scopus and Web of Science databases. Papers published from 2007 to 2021 were searched in November 2021. Of these, 52 papers were selected from the initial 788 outputs for the final analysis.

**Results:**

The final selected papers were analyzed, and the key determinants were found to be environmental, physical, cognitive, and psychosocial, which confirms the findings of previous studies. One new determinant is technological. New and effective solutions lie in understanding the interactions between different determinants of mobility, addressing environmental factors, and exploring opportunities in the context of emerging technologies, such as the integration of smart home technologies, design of accessible and age-friendly public spaces, development of policies and regulations, and exploration of innovative financing models to support the integration of assistive technologies into the lives of seniors.

**Conclusion:**

For an effective and comprehensive solution to support senior mobility, the determinants cannot be solved separately. Physical, cognitive, psychosocial, and technological determinants can often be perceived as the cause/motivation for mobility. Further research on these determinants can help to arrive at solutions for environmental determinants, which, in turn, will help improve mobility. Future studies should investigate financial aspects, especially since many technological solutions are expensive and not commonly available, which limits their use.

**Supplementary Information:**

The online version contains supplementary material available at 10.1186/s12877-023-04106-7.

## Introduction

The ageing population is an important phenomenon faced by many countries in this century. While ageing is a triumph of development, the phenomenon and its impact on the society need to be managed [1]. Mobility is crucial for active ageing as it allows older adults to maintain their independence, participate in physical activities, engage in social and community life, and access necessary resources [2]. The ability to move around freely, safely, and independently is essential for promoting physical and mental well-being, preventing falls and injuries, and reducing the risk of disability and institutionalization [3]. Addressing mobility challenges for older adults involves a multidisciplinary approach involving healthcare providers, policymakers, urban planners, and families.

Access to transportation is one of the key social determinants of health for older adults, and the lack of access is associated with negative health outcomes, including social isolation, depression, and early entry into long-term care facilities. The National Institute on Aging [2] conducted a study on the mobility of elderly Americans that emphasized the significance of accessibility in preserving the autonomy and well-being of seniors. The study suggested enhancing the accessibility of transportation systems and infrastructure to facilitate the mobility of senior citizens. The age-friendly cities framework [4] is a global initiative aimed at making cities more accessible and accommodating for older adults. It focuses on eight domains, including transportation, housing, and social participation. The universal design framework focuses on designing buildings [5], products, and environments that are accessible to people of all ages, abilities, and disabilities. The European Union has established the European Accessibility Act [6], which requires that products and services in key areas, including transportation, are accessible to people with disabilities, including seniors. The Act provides guidelines for improving accessibility in transportation systems and infrastructure to support the mobility of seniors. Accessibility and solutions to increase accessibility are directly or indirectly linked to the space where a senior needs to be mobile.

The concept of life-space mobility (LSM) assesses functional, environmental, and social factors that affect how people live their everyday lives [7]. Johnson et al. [7] showed that that socio-demographic variables such older age, female gender, and lower level of education, fear of falling, limitations in activities of daily living (ADLs) and instrumental activities of daily living (IADLs), poor performance in gait speed and muscle strength, and transportation difficulties were associated with lower LSM. Life-space mobility has been found to be a predictor of cognitive decline, hospital readmission, quality of life, and admission to a nursing home. Life-space mobility allows the initiation, adaptation, or evaluation of intervention strategies [7]. Findings from such research continue to be used by many organizations to establish better conditions. The World Health Organization’s Global Age-Friendly Cities Guide [4] provides recommendations on how to design inclusive living spaces for seniors and supports the development of age-friendly communities. The European Innovation Partnership on Active and Healthy Aging has created the Covenant on Demographic Change, which encourages local and regional governments to develop strategies for creating age-friendly environments and living spaces.

Recent technological developments are intertwined with the aforementioned strategies, programs, and actions. They are an important aspect in the search for new solutions. Porter et al. [8] describe emerging technologies as those that could exert much-enhanced economic influence in the coming several years’ time horizon. Cozzens et al. [9] showed four characteristics: fast recent growth; in the process of transition and/or change; market or economic potential that is not exploited fully yet; increasingly science-based. Finally, Smalheiser et al. state that emerging technology can be incremental, originating from its potential to change an existing industry, in addition to being radical, originating from the potential to create a new industry [10].

To sustainably improve the conditions and quality of life of seniors, it is essential to regularly identify opportunities and monitor the development and usefulness of new solutions in mobility.

The objective of the study is to identify the determinants that have had a significant impact on mobility in recent years and currently, and to identify gaps in our understanding of these factors. The study aims to highlight areas where further research is needed and where new and effective solutions are required.

As part of scoping review, the PRISMA guidelines [11] and bibliometric mapping study method [12] were used to provide a systematic and holistic review of the determinants of older adults’ mobility. Bibliometric mapping provides an overview of the state-of-the-art of scientific knowledge on a given topic. The bibliometric analysis embraces the performance analysis of contributions on specific topics, and we are using a method of scientific mapping to analyse the evolution of specific research subjects [13] in order to identify subject fields and show their progress by employing different visualization tools in research planning.

## Theoretical background

The above-mentioned studies and strategies demonstrate the importance of addressing the aspects of accessibility, living space, and technology in the context of mobility for seniors. The authors perceive the aspects of accessibility, life-space mobility, and developing technologies as intertwined, yet each of them individually significant. Accessibility, which is relevant for the given scope, is also related to the zones of the living space. Accessibility is considered to be one of the most important predictors of quality of life (QoL) for older adults [14]. The search for new solutions for the elderly is very often connected with technologies, as stated by many strategies and acts [15]. Therefore, attention will also be paid to this area within the scoping review, focusing on aspects accessibility, LSM, and technologies, to determine their objective importance in recent years. Overall, the below-described brief state of the art in the context of mobility in LSM, related accessibility, and ubiquitous technologies, shows how the field is perceived in the context of this paper.

### Mobility and zones of living space

Zones of living space for the elderly are designed to meet their specific needs and support their mobility. These zones typically include residential areas that are designed to be accessible, with wide sidewalks, low curbs, and other features that make it easier for older adults to get around. In addition, these zones may also include essential services such as healthcare facilities, grocery stores, and public transportation that are easily accessible to older adults. The idea behind designing zones of living space for the elderly is to create an environment that supports their mobility, promotes their independence, and enhances their quality of life [16]. Webber et al. [16] introduced a model defining seven zones of living space for older adults: room, dwelling space, close neighbourhood, vicinity, service community, surrounding area, and the world. The cognitive, emotional, physical, environmental, and economic elements that determine mobility vary among zones and are heavily influenced by gender, culture, and individual biography. Similarly, Burlano and Cusano specify four types of neighbourhoods according to local accessibility and possibilities of travel to distant places [1]. Different zones are associated with different types of spatial mobility. Many authors emphasize the benefits of physical movement in maintaining physical and mental health and the positive impact of soft mobility (cycling, walking, etc.) in alleviating diseases. Cao et al. confirm that engaging in outside activities can improve older adults’ well-being. Mobility declines with age mainly due to health constraints and changes in activity patterns [17], which increase the risk of sedentarism and hypomobility. Spatial mobility is not only intended to fulfil physiological needs but is also an important part of social cohesion [18], social participation [19], and the basic condition required to maintain older adults’ independence and self-reliance [20]. Many studies [21–24] recommend engaging in out-of-home travel regularly to interact with friends and the broader community to reduce social isolation.

### Accessibility

Accessibility studies reflect spatial mobility opportunities and conditions. Geurs [25] defined accessibility as “the extent to which the land-use transport system enables (groups of) individuals or goods to reach activities or destinations by means of a (combination of) transport mode(s)”. It is evaluated using different measures. Geurs specified the following four basic types of measures: infrastructure-, location-, person-, and utility-based.

Location-based measures focus on potential and connectivity, but they typically do not take into account individuals’ behaviour, capacity restrictions, return routes, chaining of destinations, or temporal variability. Accessibility is measured from the perspective of an individual’s capacity to make use of resources within the context of their normal routine and time restrictions [26]. Such measures may well address the richness of older adults’ lives, but they rely heavily on data input [27]. Accessibility includes the evaluation of

1. the availability of destinations and required activities,
2. physical access and walkability, and
3. functionality of transport systems, including affordability and acceptability (safety, information support, etc.) [28].

All these components are highly influenced by personal factors. The qualitative assessment of accessibility (modelled accessibility) significantly differs from perceived accessibility evaluated by personal survey tools.

Accessibility indexes and mode choice studies are receiving more attention from researchers. “Aging in place” refers to the desire of the elderly to remain in the homes and communities they have been in for the longest possible period of time after retiring [21]. It is not unusual for individuals to choose the same neighbourhood where they have spent most of their adult years as a retirement location, especially in suburban settings.

### Mobility opportunities in relation to emerging technologies

Elsy [29] has observed that technologies are developing towards an environment where humans, robots, or artificial intelligence (AI) coexist and work together to improve the quality of life by offering specialised services for the needs of diverse users. This enables an increased level of autonomy by proactively collecting data from the environment, making decisions, and providing services for humans. Older adults’ healthcare solutions include (1) connecting and sharing information in the network between users of medical data, including medical check-up records and treatment and care records; (2) providing remote medical care services; (3) and using AI and robots in the care facilities to support community independence.

However, existing new mobility options have left a large population of harder-to-serve older adults out of the new mobility revolution, so that they are unable to access its benefits. Those with more limitations (physical, cognitive, multiple disabilities, financial, technology access/understanding) or certain specific needs (transportation that accommodates mobility aids, low-income, rural location) often face barriers:

1. Using emerging transportation technologies requires smartphone and internet access, as well as the ability to make online payments, which can be challenging for many older adults who may lack even a basic level of tech fluency and comfort.
2. The vehicles can be difficult to enter and exit and may not easily accommodate walking aids or wheelchairs, which are used by approximately a quarter of all older adults.
3. Current ride-hailing and projected autonomous vehicle (AV) services primarily focus on curb-to-curb service, while many older adults need door-to-door or hand-to-hand service due to challenges such as identifying the vehicle when it arrives; stowing and retrieving bags or mobility aids; finding the correct door at complex locations such as hospitals or shopping centres; and physically navigating busy streets and sidewalks [30].

This systematic review will examine and identify mobility limitations in the context of technology development, highlighting opportunities for further development.

## Method

### Study design

Traditionally, the evaluation of older adults’ mobility is based on self-reporting and subjective indicators. Although such indicators are usually considered appropriate to characterize the individual mobility of seniors, they are unsuitable for effectively assessing the situation on a larger scale. To overcome this limitation, we conducted a scoping review using bibliometric mapping and clustering. This approach provides an independent view of the structure of scientific discussion on the topic and allows for the identification of common groups of problems and neutral assessment/quantification of co-occurrences and associations, free from the researchers’ biases.

We followed the five-step research process outlined by Arksey and O'Malley [31] as the methodological framework for a scoping review. Before making a final decision, the research questions were determined and the significance of the study was established. Data were then collected, summarized, reported, and eventually visualised. To ensure a comprehensive and methodical overview of the factors affecting mobility in the elderly, we employed the above-mentioned framework for the scoping review approach in accordance with PRISMA standards [11] and the bibliometric mapping study method [12].

To minimize bias and potential for errors, two groups of reviewers (first group: Krejcar, Maskuriy, Abu Bakar, Selamat and second group: Maresova, Truhlarova, Vitkova, Joukl, Horak) independently coded each study with a present form or data extraction tool. They collected study details, such as methods and design, participants, setting, interventions, and results.

#### Data extraction and study quality evaluation eligibility

Eligible works were sorted by the researchers who had independently reviewed the studies. Each paper was examined in terms of its title, the author(s), publication type, and language. Studies meeting the following specific set of criteria were selected for further review:

1. Published until 2021
2. Focusing on studies related to older adults
3. Focusing on studies analysing life space environment and mobility determinants

Publications were excluded if they met any of the following criteria:

1. If the mobility was understood as a senior’s willingness to move
2. If mobility was perceived as a part of recreational travel (holidays, etc.)
3. If seniors were a minority group in the research and their perspective was analysed only marginally
4. If the focus was on aspects of population distribution in specific urban areas by age
5. If mobility was examined as one of the factors at the end of the research objective (mobility, perception of loneliness, evaluation of the type of accommodation, etc.)

We collected bibliometric studies of papers published from 2007 to 2021 in Scopus and Web of Science (WoS) as of 16 November 2021. The starting year was set at a minimum number of 10 papers per year, which was reached in 2007 with 11 papers. The number of papers increased over time, with 108 papers in 2021 in WoS. The search used the following keywords: (((spatial mobility) and ((elderly) or (senior) or (geriatric) or (older) or (aging)) and (home or model))) in all fields. For WoS, only the Social Sciences Citation Index (SSCI) and Science Citation Index Expanded (SCI-E) were selected. The search criteria were also limited in terms of publication type, and this to Article, Early Access, or Review Article. The number of results obtained is listed in Table 1. The majority of papers were published in the International Journal of Environmental Research and Public Health [17], Journal of Transport Geography [16], Population Space and Place [16], Plos One [14], and ISPRS International Journal of Geo Information [9]. Eight articles were published in Scientific Reports, Transportation and Transportation Research Record. The papers covered various WoS Categories, with the highest numbers in Geography [32], Public Environmental Occupational Health [33], Transportation [34], Environmental Sciences [35], Environmental Studies [36], and others.

**Table 1:** Article distribution according to databases and publication type

Database	All	Articles	Early Access/In Press	Review
Scopus	252	240	8	8
WOS	536	520	11	5
TOTAL	788	760	19	13

### Data selection

**Fig. 1:**
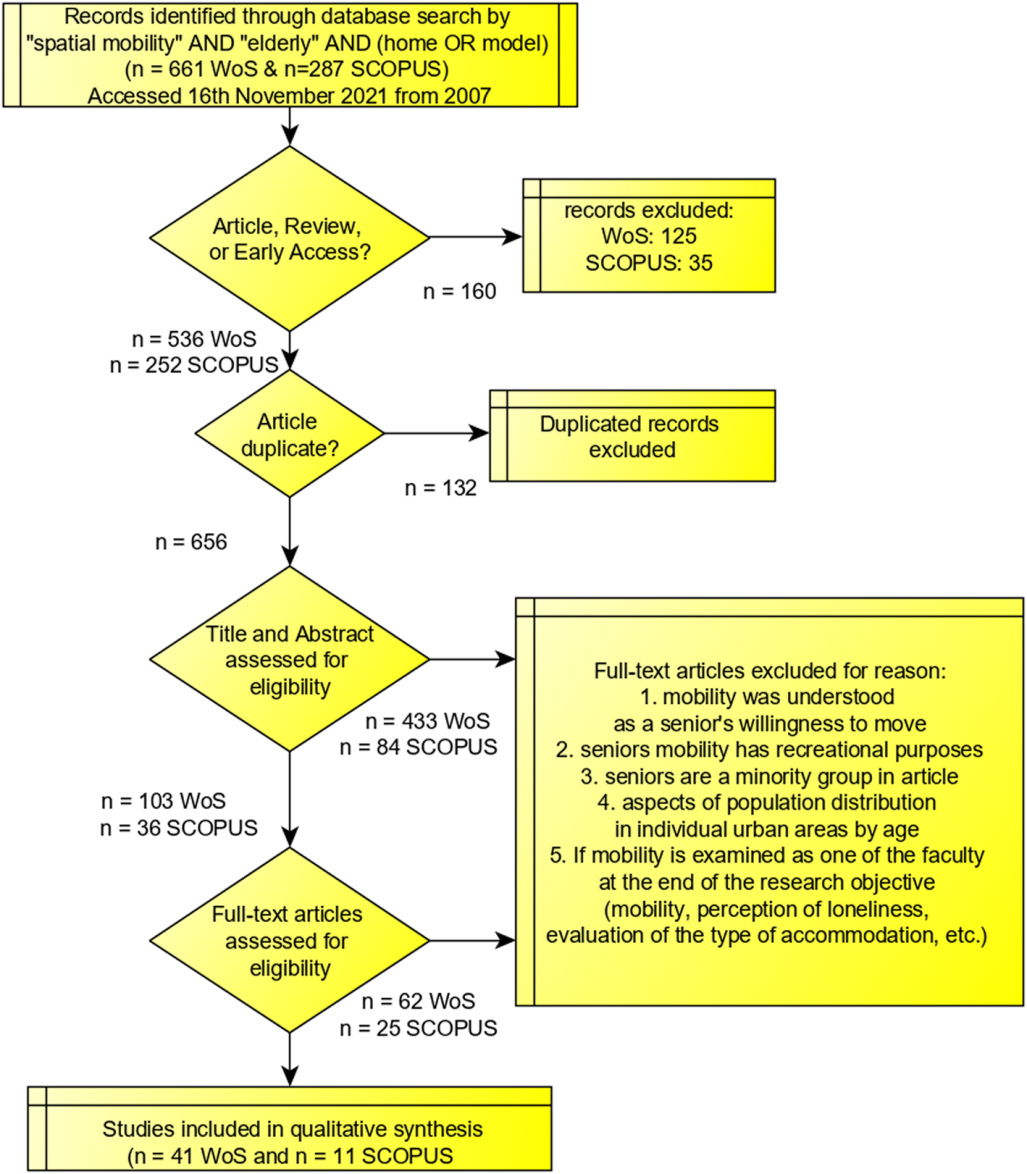
Systematic review and meta-analysis following the PRISMA flow of articles with the final selection

The final data selection process from the search result set was conducted following the PRISMA framework [11], as shown in Fig. 1.

A total of 760 publications were initially identified through the search on WoS and Scopus using the search term “(((spatial mobility) and ((elderly) or (senior) or (geriatric) or (older) or (ageing)) AND (home OR model))”. This resulted in 536 papers from WoS and 252 papers from Scopus. After removing duplicates, the number was reduced to less than 132 papers. Due to the lack of granularity in the keyword search and the inability to narrow down the search topics based on keywords and focus areas, a manual scoping process was employed to identify the most relevant papers. Many of the initially identified works were found to be irrelevant, resulting in the final selection of 139 articles with correct and suitable content based on a deeper analysis of their abstracts. Additional 87 papers were excluded from the selection, finally leaving a total of 52 fully synthesized records for analysis. Details of these analysed records are summarised in Tables 6 and 7.

### Bibliometric mapping

Bibliometric mapping is a technique that provides a visual representation of the performance of published studies, describing the perception and information related to a specific research area and the real possibilities of mobility. 

This technique, introduced by Garfield [37], involves the application of a set of mathematical methods and statistics to analyse and measure publications. It aims to establish qualitative and quantitative changes within a given scientific research topic and detect publication profiles and trends within a discipline [38].

Bibliometric analyses use bibliographical material to organize and analyse information in a particular field, providing valuable insights for experts assessing scientific activity. These analyses help in understanding the past, identifying advances in research, and enhancing future investigations.

Scientific mapping, as used in analysing the evolution of specific research subjects [13], enables the identification of subject fields and demonstrates their progress using various visualization tools in research planning.

VOSviewer, a free software solution developed by van Eck and Waltman [39], is employed for building and visualizing bibliometric maps. It offers the major advantage of selecting and classifying scientific documents, particularly for constructing conceptual maps.

## Results

### Bibliometric mapping

#### Results according to countries

**Fig. 2:**
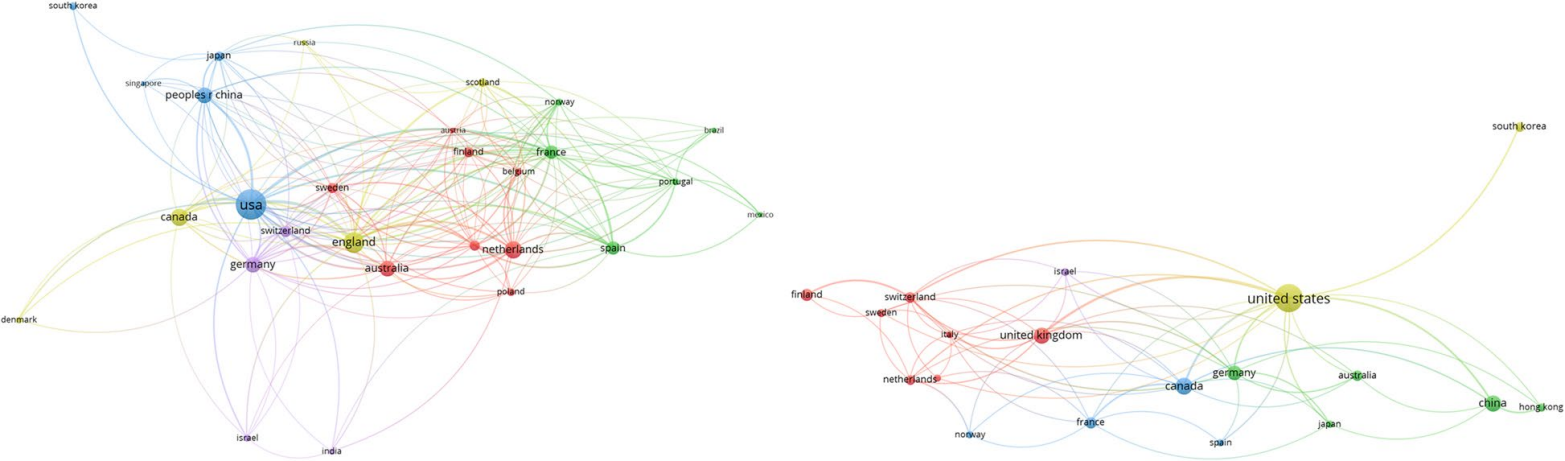
Co-authorship of countries in Web of Science (left) and Scopus (right)

First, the analysis of countries where research on the mobility of seniors is focused was conducted. Figure 2 illustrates the co-authorship of countries that have published at least 5 papers related to the search defined in “Study design” section in the respective databases from 2007 to 2021.

Table 2 shows the co-authorship data for both databases. Link strength is a metric that captures the number of other keywords with which the specified keyword co-occurred (i.e., was linked) in the reviewed database [39]. In Web of Science, out of 70 countries, 29 meet the thresholds. The United States published the most with 176 documents, followed by England [40] and the Netherlands [41]. In Scopus, out of 53 countries, 19 meet the thresholds. The United States still published the most in Scopus with 76 documents, followed by Canada [28] and China [27]. The United States has the strongest connection to the keywords searched, with links to a maximum of 24 countries and a links strength of 117, making it the leader in the research topic. The last row in Table 2 represents the results for EU countries, which show higher values compared to the United States.

**Table 2:** Co-authorship of countries

Web of Science					Scopus				
**Country**	**Doc**	**Citations**	**Link Strength**	**Link**	**Country**	**Doc**	**Citations**	**Link Strength**	**Link**
United States	176	4056	117	24	United States	76	2295	37	15
England	76	2541	105	25	Canada	28	2274	17	10
Netherlands	56	889	51	17	China	27	354	11	5
Canada	54	2637	36	14	England	27	1576	19	10
Australia	48	777	40	13	Finland	15	367	6	3
Germany	45	778	54	20	France	13	258	10	8
China	45	479	33	14	Australia	12	223	6	5
France	35	771	71	22	Switzerland	11	1081	16	9
Spain	34	547	44	16	Netherlands	9	410	11	8
Switzerland	22	1125	36	13	South Korea	9	114	3	1
Finland	21	488	21	16	Hong Kong	7	89	5	3
Sweden	21	397	32	15	Israel	7	1190	7	6
Italy	20	1325	49	20	Sweden	7	285	6	6
Japan	17	228	23	15	Italy	6	1090	9	8
Scotland	14	364	25	12	Poland	6	37	5	5
EU	236	4804	-	-	EU	98	4144	-	-

**Fig. 3:**
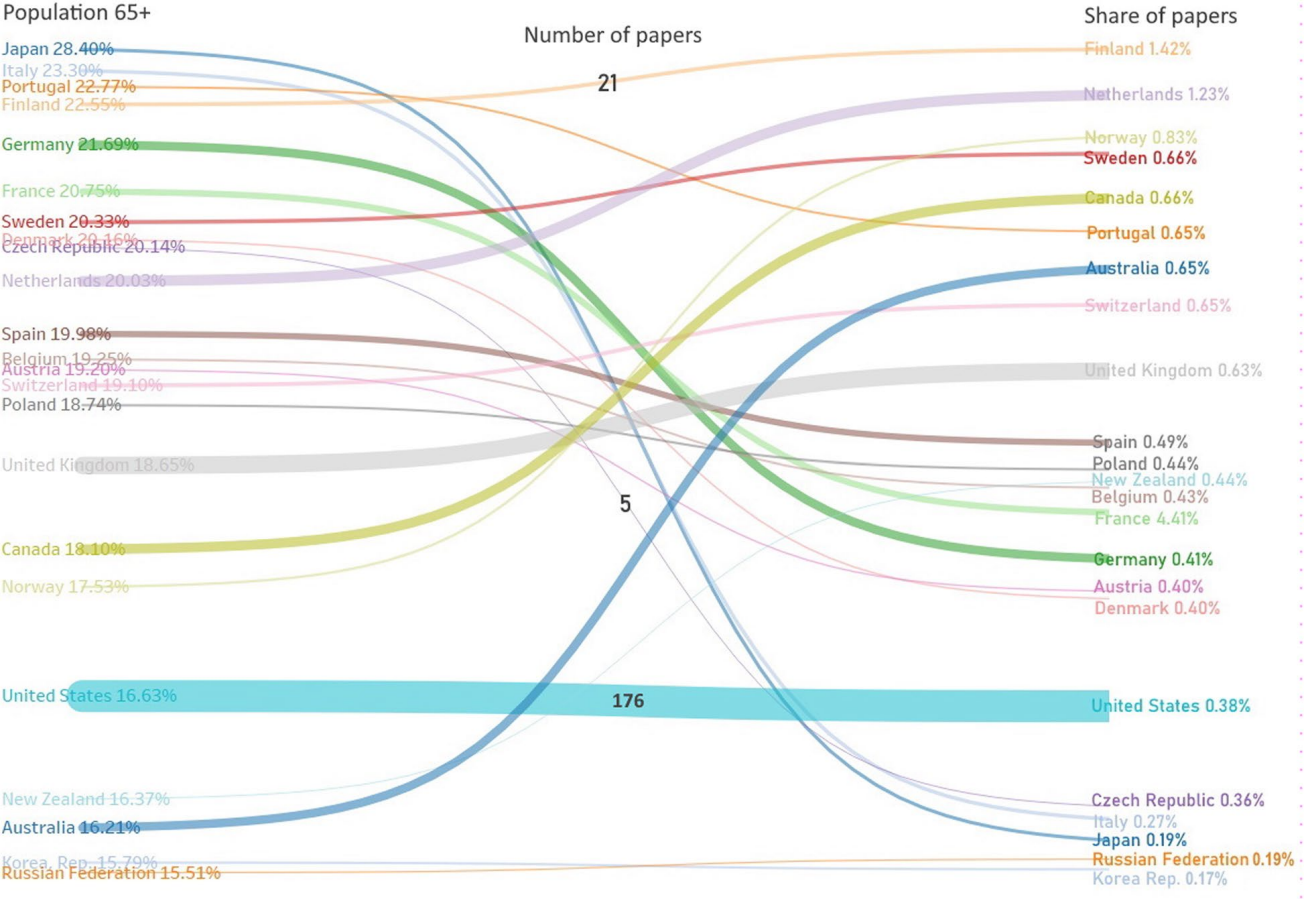
A Sankey diagram of the connection between the shares of seniors (Population 65 + ; left part) in a selected country [42] and the share of the searched papers from the total publishing activity of the given country in the period from 2007 to 2021 in WoS

Figure 3 shows that the correlation between the population of people aged 65 + in a country and the number of papers on spatial mobility is weak. Some countries (Japan) do not investigate the issue of spatial mobility, while the Nordic countries do. The United States is an exception, where the publishing activity is proportional to the senior population.

#### Cluster analysis of keywords

**Fig. 4:**
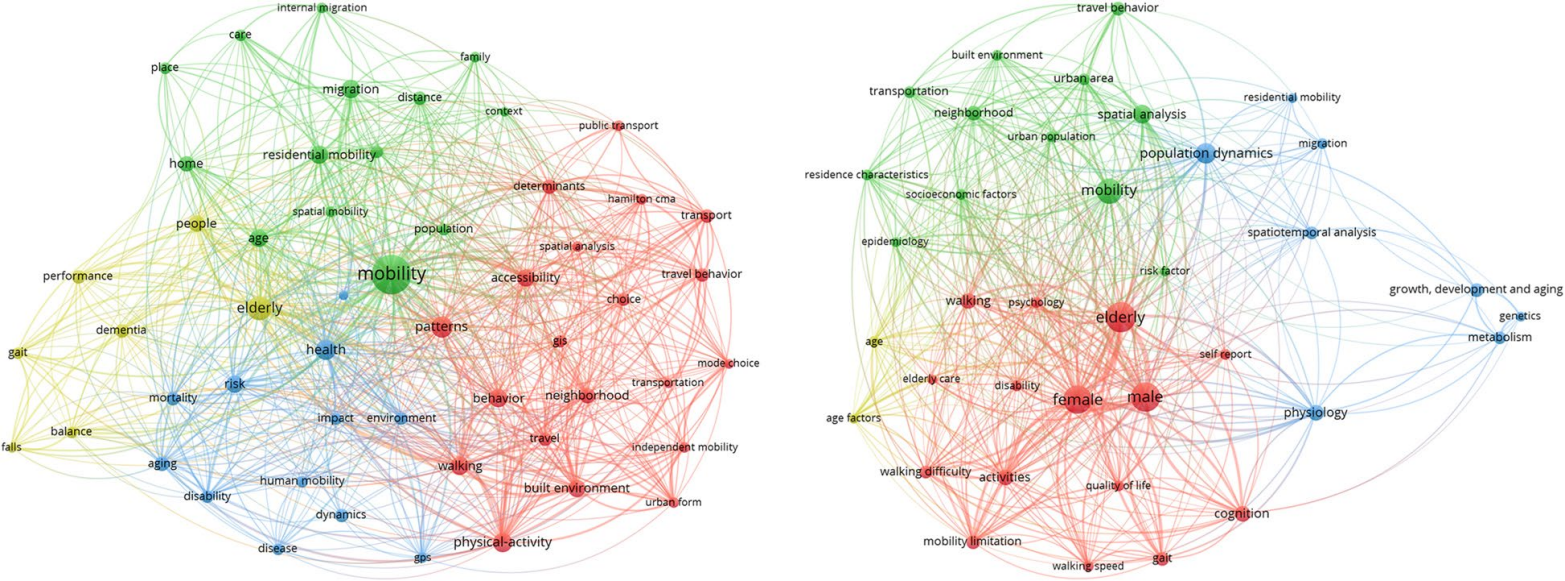
Co-occurring keywords in Web of Science and Scopus clustered into 4 clusters with different colours (red, green, blue, yellow)

Using the terms “spatial mobility” and (“elderly” or “senior” or “geriatric” or “older” or “ageing”) AND “(home OR model)” in Web of Science and Scopus from 2007 to 2021, we were able to locate, evaluate, and analyse the published works to determine their structure and trends. The most frequently used keywords for conducting keyword searches were identified, as shown in Fig. 4 generated by VOSviewer. In VOSviewer, the overall strength of each keyword’s linkages with other terms and its frequency of occurrence were calculated. Co-occurrence analysis was performed to establish the proximity of keywords within articles (name, abstract, or keyword set) and focus on the topic of research [43]. Each individual circle represents one keyword, with the size of the circle indicating the number of occurrences of the specific keyword. The links between these keywords (circles) represent papers where both keywords occur, and the thickness of the links represents the strength of their connections (links).

The graph of the WoS collection shows an intersection of four clusters or domains. These clusters include the individual performance cluster (yellow), which consists of keywords related to balance, dementia, gait, etc.; the basic health and social cluster (blue), likely formed by sociological papers; the mobility cluster (green); and an overlap between the mobility cluster and the environment cluster (red). This indicates three main drivers for older adults: individual performance, health and social factors, and mobility connected with the environment and social aspects.

The WoS collection contains more medical and geriatric papers, often related to LSM, as reflected in the individual performance cluster.

Co-occurrence refers to the proximity of a keyword in the title of the research work, the abstract, and its list of keywords [44]. 

Tables 3 and 4 show the most commonly associated keywords within the identified clusters, considering keywords that co-occurred in at least 10 articles. In Web of Science, out of a total of 3716 keywords, 70 met the threshold, and 5 clusters were identified from the top 50 keywords. In Scopus, although the number of publications was much lower than in Web of Science, the total number of keywords found was similar, with a minimum of 10 occurrences of a keyword. Overall, 3221 keywords were found in Scopus, 65 keywords met the threshold, and 3 clear clusters were identified. The top co-occurring keywords in Web of Science, apart from the main keywords and demography, were ‘pattern’ (43 occurrences), ‘health’ [45], ‘physical activity’ [42], ‘migration’ [38], and ‘behavior’ [37]. In Scopus, the top co-occurring keywords were ‘population dynamics’ (41 occurrences), ‘spatial analysis’ [38], ‘activities’ [26], ‘cognition’ [25], ‘physiology‘ [25], and ‘walking’ [24]. The co-occurrences and connection strengths to the keywords are shown in Table 5.

**Table 3:** Clusters of keywords that co-occurred in Web of Science under the keyword search related to it. Only the top 50 keywords out of a total of 3716 were considered

Cluster	Keywords
1	Patterns, physical-activity, behavior, walking, accessibility, built environment, neighborhood, determinants, transport, travel behavior, travel, choice, GIS, Hamilton CMA, spatial analysis, urban form, independent mobility, public transport, mode choice, transportation
2	Mobility, migration, residential mobility, age, home, distance, gender, care, place, population, spatial mobility, family, internal migration, context
3	Health, risk, aging, mortality, dynamics, disability, environment, human mobility, disease, impact, GPS, quality
4	Elderly, people, dementia, performance, gait, balance, falls

**Table 4:** Clusters of keywords that co-occurred in Scopus under the keyword search related to it

Cluster	Keywords
1	Elderly, male, female, activities, cognition, walking, mobility limitation, gait, walking difficulty, psychology quality of life, walking speed, disability, self-report, elderly care
2	Mobility, spatial analysis, neighborhood, travel behavior, urban area, transportation, residence characteristics, socioeconomic factors, built environment, urban population, epidemiology, risk factor
3	Population dynamics, physiology growth, development and aging, metabolism, spatiotemporal analysis, migration, genetics, residential mobility
4	Age, age factor

**Table 5:** Clusters of keywords that co-occurred in Web of Science under the keyword search related to it with the number of occurrences (O) and total link strength (TLS)

**Web of Science**											
**Cluster 1**			**Cluster 2**			**Cluster 3**			**Cluster 4**		
**Keyword**	**O**	**TLS**	**Keyword**	**O**	**TLS**	**Keyword**	**O**	**TLS**	**Keyword**	**O**	**TLS**
patterns	43	103	mobility	146	371	Health	41	128	elderly	55	199
physical activity	35	145	migration	33	68	Risk	30	84	people	27	81
behavior	32	70	residential mobility	32	66	Aging	20	74	dementia	18	46
walking	32	137	age	28	76	Mortality	19	51	performance	17	39
accessibility	30	83	home	22	51	Dynamics	17	19	gait	14	41
built environment	28	125	distance	17	45	Disability	16	58	balance	13	44
neighborhood	23	94	gender	16	45	Environment	16	53	falls	10	37
determinants	21	68	care	13	39	human mobility	14	18			
transport	19	67	place	13	19	Disease	13	26			
**Scopus**											
**Cluster 1**			**Cluster 2**			**Cluster 3**			**Cluster 4**		
**Keyword**	**O**	**TLS**	**Keyword**	**O**	**TLS**	**Keyword**	**O**	**TLS**	**Keyword**	**O**	**TLS**
activities	26	201	mobility	66	260	population dynamics	41	165	age	13	108
cognition	25	135	spatial analysis	33	156	Physiology	25	139	age factor	10	82
walking	24	152	neighborhood	23	126	growth, development and aging	18	29			
mobility limitation	19	154	travel behavior	19	68	metabolism	16	38			
gait	18	115	urban area	15	81	spatiotemporal analysis	16	45			
walking difficulty	16	138	transportation	14	62	Migration	12	39			
psychology	13	111	residence characteristics	12	108	Genetics	10	22			
quality of life	12	79	socioeconomic factors	12	98	residential mobility	10	24			
walking speed	12	83	built environment	11	73						
disability	11	84	urban population	11	66						

Some keywords, such as ‘Accessibility’ and ‘mobility’, can be seen as factors or determinants related to the keyword search, as confirmed in Tables A1, A2 and A3 in the annex, which show that the top 10 keywords are most closely linked to the keywords ‘mobility’, ‘Accessibility’, and ‘spatial mobility’ in Web of Science and may serve as determinants for the keywords search. For example, ‘patterns’, ‘health’, ‘migration’, ‘built environment’, ‘transport’, ‘risk’, ‘disability’, ‘neighborhood’, ‘physical activity’, and ‘distance’ were linked to ‘mobility’ in WoS.

In Scopus, ‘mobility’ and ‘spatial analysis’ have similar weights as in Web of Science, as confirmed in Tables A4 and A5 in the annex. Both terms indicate high overlap in the list of keywords, particularly in keywords with higher total link strength (TLS). However, spatial analysis is linked with the urban environment (keywords: ‘urban area’, ‘urban population’), socioeconomic factors, and risk factors.

**Fig. 5:**
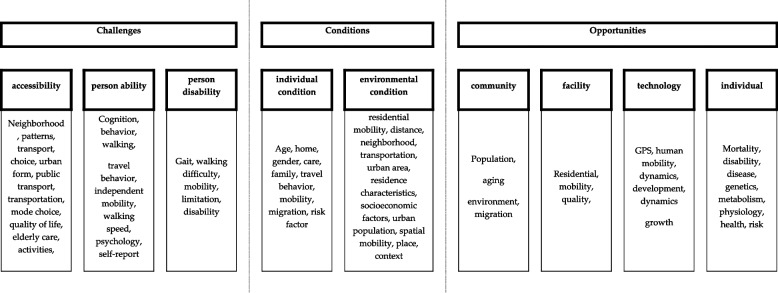
Thematic mobility clusters based on the co-occurring keywords identified with VOSviewer

Preliminary clustering conducted with VOSviewer provided insights into the relationships, occurrences, and co-occurrences of keywords and helped to select those that represent the main common influencing factors. This enabled the proposal of a final clustering/categories in Fig. 5. Three comprehensive clustering distributions with nine sub-clusters were selected and named (Fig. 5). The first cluster encompasses a wide range of challenges related to accessibility and ability. The second cluster focuses on individual and environmental conditions that influence determinants of mobility. The third cluster reflects opportunities in the community, facility, technology, and individuality that influence perception, accessibility, and the improvement of spatial mobility.

### Determinants of elderly mobility

Table 6 provides an overview of selected publications that focused on mobility determinants in older adults, including their main purpose, methods used, results, and constraints. A qualitative approach was employed in the research in this section. The keywords clustered below are already included within the thematic clusters, which encompass studies from the areas of accessibility, personal ability and disability, individual and environmental conditions, community, facility, and technology.

**Table 6:** Summary of 52 targeted papers

Cluster	Source	Method	Findings	Limitations
Challenges	[46]	Seven persons with dementia (PwD) and eight people with normal cognition (CTLs) over the age of 65 were tracked for four weeks using a GPS device. This was done when they went out of their houses during a period of four weeks	When compared to cognitively intact CTLs, PwDs engaged in more medically related and less sport-related activities, while neurotypical CTLs walked more and spent more time outside at nighttime	Limited generalization because of a small sample size
	[47]	Measures of planning (HOTAP test), spatial working memory (Grid-Span test), and visuospatial attention were used to test cognitive abilities (Attention Window test). An instrumented version of the Timed Up-and-Go test was used to measure the ability to move around (iTUG). Smartphones with accelerometers and GPS were used to measure mobility over a one-week period.	The assessments of mobility were whittled down to four orthogonal components: the factors indicating “real-life mobility”, “sit-to-stand transition”, and “turn” connected with fewer cognitive measures, while the factor indicating straight gait linked with just one cognitive indicator	Only limited number of factors were analysed
	[48]	Tests: gait speed, Five Times Sit to Stand, Four Square Step Test (FSST), and Dynamic Gait Index (DGI)	There were significant connections reported between increased regional grey matter volume (GMV) in many areas, most notably the parietal and temporal lobes, and better performance in gait speed, distance gained in the digit span test (DGI), and forced swimming speed test (FSST)	Limitations are not described
	[45]	Regression models	Cognition, logic, and response time were all shown to have a substantial impact on a person’s life space, and reasoning ability was found to be the most predictive. In addition to the standard cognitive tests, assessments of everyday function showed strong relationships with living space	The findings indicated the necessity of doing more research over a longer time span
	[36]	Global positioning systems and location kits were used to track the participants for 28 consecutive days (GPS)	The outside-of-home time spent by seniors with cognitive impairments has a severely constrained spatial range	
	[49]	There were 260 neighbourhood men and women aged 75–80 who responded to a mail survey evaluating physical mobility (utilising the Life Space Questionnaire) and mental wellbeing (using the SF36 Health Related Quality of Life Profile)	It has been shown that elderly women are more prone to face a lack of mobility and confined life space, which makes them more sensitive to social isolation	
	[50]	For up to seven days, 393 participants between the ages of 18 and 65 were tested using the Patient Health Questionnaire (PHQ-9) and cellphones equipped with global positioning systems (GPS). A number of different linear regressions have been performed	The 50-m buffer used in the fully adjusted residential and mobility-based models revealed a negative correlation between depression symptoms and proximity to green space	Work on the environment and psychological health necessitate further investigation of dynamic sensitivity assessment methods
	[51]	More than 100 older adults were observed in their homes for a cross-sectional research. Multiple logistic regression was used to discover characteristics that could distinguish three degrees of frailty	Gait speed (area under the curve, AUC = 0.802), hip sway (AUC = 0.734), and steps/day (AUC = 0.736) were the most sensitive parameters for the identification of prefrailty	
	[52]	Cross-sectional research that took account of 118 multimorbid older adults that presented with cognitive issues	Low and high life-space mobility (LSM) can be distinguished by a crucial value on the receiver-operating characteristic curve (ROC) People with cognitive impairment can benefit from tailoring therapy to their specific LSA-CI scores and tracking their progress over time	Further investigation must focus on GPS as the subsequent best feasible practical method for determining LSM, which is a drawback of this work
	[53]	For this study, researchers drew on the medical records of 13,400 people in Quebec who were diagnosed with schizophrenia during 2001–2002	The resulting model shows that at the individual basis, men, wonder patients, and physiological comorbidity enhance the risk of early home movement, while older adult patients are less prone to relocate sooner	Diagnostic codes stored in administrative systems can have an impact on estimating accuracy
Conditions	[54]	Customized mobility measures for Montreal Island, based on location and individual data on travel patterns that have been geocoded	Seniors and non-seniors, as well as those who own or do not own a car, have vastly different levels of access to transportation. The focus of this study was on the possible accessibility of the information	Follow-up studies might use the findings from this study to choose case studies of real access and utilisation of health care services and associated health outcomes
	[55]	Six community-dwelling older individuals completed a 14-day self-reported activity journal to collect data. The examination of the data includes 1453 occurrences	To better educate themselves and their patients, doctors and researchers may use workflows to define the everyday activities of older adults and develop education and preventive plans that are specific to each person’s level of activity	Data is from a sample which is small and spans only over two weeks
	[56]	Non-dementia community-dwelling seniors were researched in 571 cases. A total of 303 individuals reported incidents of life-space constrictions over an overall follow-up period of 4.3 years	A higher chance of life space recovery was seen in people who had a valid driver’s licence. Older adults with a valid driver’s licence were less likely to report life-space restriction and more likely to recuperate from living restriction if such an event happened.	Even though there was a link between driving status and reports of life space being too small, the authors could not determine whether it was because of the group of people who had never had a valid driver’s licence or the group of people who had a valid licence at some point but did not renew it at least 12 months before our baseline interview.
	[57]	Data from the Hamilton CMA in Canada and multi-level models are used in this study to analyse the variables that influence travel distance, with a particular focus on demographic ageing issues	Whereas this age impact is apparent in all modes of analysis (driving a car, riding in a car, and using IRIS), driving a car has a far more pronounced effect. When it comes to a vehicle driver’s distance travelled, a neighborhood’s mix of business and residential properties had a negative correlation with distance travelled exclusively in the case of the car driver	The evaluated variables were restricted to a small number. According to this study, it would be interesting to find out if older adults have similar domestic constraints (such as taking on the responsibility of caring for grandchildren) to women (for example, health limitations) or other factors (such as the loss of a licence, for example), that put them at the same degree of limit to move much further
	[58]	Analysis of data from the Life-Space Mobility in Old Age cohort study at the beginning of the research period. Participants’ houses for structured interviews. Participants were older adult individuals (*N* = 848) who live in a community. Mobility was measured in one’s environment (Impact on Participation and Autonomy questionnaire subscale, Life-Space Assessment; University of Alabama, Birmingham Study of Aging), as well as short physical performance battery	The average life-space mobility score was 64.0 out of 100 For those who had poor physical performance and had a more limited feeling of autonomy, their life-space mobility was restricted by around one-third of the variance. Outside, a person’s feeling of self-determination was influenced by their physical performance in turn	The research of the participants’ level of variability may have been raised by administering a more taxing physical function test or a discriminative assessment of their sense of self-determination
	[59]	Statistical analysis, regression, correlation	Larger living quarters were linked to higher self-reported impairment levels. According to generalised logit models adjusting for demographics and season, a wider living space was related with reduced vision disability, greater levels of lower extremity motor function, global awareness and social participation as well as with personality and a sense of life’s meaning. As a health indicator in old age, older individuals’ range of environmental mobility may be a valuable supplement to measurements of handicap	It was only possible to evaluate the correlations of life space since the studies were cross-sectional. As a result, volunteering participants from the set of community-dwelling individuals may have had bigger living spaces than the overall population
	[60]	Data come from the 5-year follow-up of the Advanced Cognitive Training for Independent and Vital Elderly (ACTIVE) Study (*N* = 2,765; mean age = 73.6; 75.8% women; 73.7% White). Life-space constriction was defined as not traveling beyond one’s town. A series of logistic regression and Cox proportional hazard models were used to estimate risk for incident life-space constriction by race and gender.	At the outset, life-space restriction was more common among people of colour and women. At follow-up, women were more likely than men to have experienced incident life-space compression. Compared to Whites, Blacks had a decreased chance of life-space restriction over time	Towns were defined in a small sample size and may have differed from respondent to respondent as well as geographically (e.g., rural vs. urban areas)
	[61]	For a 2-year period, 848 persons aged 75 to 90 were questioned in their homes and evaluated using generalised estimation equation models (GEEs)	The findings emphasise the necessity of ensuring that older adults have access to out-of-home mobility options to preserve their quality of life	Self-evaluation of quality of life (QOL) might fluctuate. As a result, we cannot say with certainty when the shifts in QOL occurred. Because certain baseline factors may have changed over time, as different chronic illnesses, for example, we cannot discount the potential that these alterations reflect the connection between mobility change and quality of life
	[41]	Home-based interviews at the start of the research, followed by phone interviews after 2 years. The two were a part of the Life-space mobility in old age cohort study’s long-term analysis. A total of 755 residents of Central Finland aged 75–90 years were included in the study. Results: ADL disability status and LSA score (range 0–120)	Restrictions and declines in mobility in old age may be early indicators of increased vulnerability to impairment, as per the findings of this work	Those older adults who were more frail were underrepresented in the sample, and those who were included had a slightly increased likelihood of dropping out during the follow-up, which is a condition that is frequent in studies on ageing
	[62]	We used multivariable logistic regression analysis on the binary outcome transportation walking (vs. motorised transportation) to evaluate the association with perceived mobility facilitators in the neighbourhood. The results showed that walking was more likely to be used than motorised transportation.	According to the findings of this study, older people’s transportation preferences appear to be influenced by their perceptions of the environmental qualities of the surrounding neighbourhood. It may be feasible to boost the number of older adults who walk for transportation on a population level by taking environmental measures or by providing information to older adults about the alternatives available to them in the surrounding area	The cross-sectional study design does not permit inferences of causality, and the results may be influenced by residential self-selection, limited sample size, and variables with small subgroups. In addition, the study relied on self-reporting, and some of the measures, including physical activity and the outcome variable, were rather crude.
	[35]	Central Finland’s Jyvaskyla and Muurame municipalities provided the participants for this likely cohort research with a follow-up period of two years. The subjects were 75–90 years of age and lived in community settings. The research was conducted in the context of the Life-Space Mobility in Old Age study (LISPE), which was carried out during the years of 2012 and 2014	Even after accounting for several measures of health and functioning, the results maintained their statistical significance. According to the findings of this study, providing assistance to senior citizens in the pursuit of personally meaningful objectives throughout their lives may add to a bigger life space while also, as a result, to a higher quality of life in old age	There is a possibility that the participants had other objectives in mind, but they did not state those in the interview. Since we do not have any data on the participants’ objectives at the follow-up, we are unable to determine whether or not they have evolved
	[63]	A FitBit Surge was used in three days to monitor thirty subjects	A substantial correlation was found between the number of hours spent using GPS and levels of cognition. The results of this study point to the possibility that GPS watches might be used to continually monitor changes in functional health in order to provide information for preventative efforts	Sample size is too small, alongside the time period of the test
	[34]	The participants in the research ranged in age from 75 to 90 years of age, and their step counts and total activity were monitored using an accelerometer (a Hookie AM20 Activity Meter) over 7 days	The Life-Space Assessment (LSA) questionnaire was utilised to do an analysis of life-space mobility. Overall, sixteen percent of people who moved around independently had a life-space region that was limited to the neighbourhood. Participants whose life spaces were more limited were less physically active, and around seventy percent of them had unusually low values in daily step counts (615 steps) and moderate exercise time (6.8 min). A favourable correlation was found between higher step counts and total activity time and life-space mobility.	There is a need for prospective research to shed light on the chronological sequence of low levels of physical activity and restrictions in the ability to move freely in one’s life space. The sensitivity of an accelerometer in older persons walking with assistance devices is yet unknown. Because of this, it is probable that the physical activity measurements utilised in this study overestimated the amount of activity engaged in by those who routinely use assistive devices such as walkers. The life-space mobility score takes into consideration whether the individual relies on human assistance or assistive equipment in order to reach a particular level of life-space
	[64]	In addition to an investigation of the mean journey characteristics, a sequence alignment approach is utilised in this process	Walking was the form of transport that was most common for persons of senior age, regardless of the type of trip purpose. The vast majority of activities are carried out in the immediate vicinity; trips further afield are often reserved for medical needs and trips to see relatives. Second, variables such as older age, living alone, a high degree of physical handicap, a poor level of education, large distances from home to the nearest public transit, having paid job, and an inability to drive are all things that prevent senior people from travelling.	The following are some of the limitations of the study as well as opportunities for more research: a) In order to achieve more valuable policy implications, a more strict statistical causation is necessary between the structural motif information and the sociodemographic data; b) An in-depth study of the policy implications should be performed.
	[65]	Cross-sectional postal survey was carried out, logistic and linear regression models were used.	The only factor that was linked with the total quantity of walking in minutes per week was residential density. There were shown to be moderating effects of gender, age, and the utilisation of walking aids. It would suggest that making improvements to the physical environment might be a viable chance to incentivize and allow older persons to walk for transportation	There is a source of uncertainty in the fact that older adults have a tendency to exaggerate their level of physical activity when answering the questionnaire. Additionally, it is possible that the respondents did not adequately differentiate between walking for transportation and other types of walking activities.
	[66]	We used data from the RECORD Cohort Study collected in 2011–2012 in the Paris metropolitan area, France. A sample of 2062 individuals was investigated, Multilevel linear regression was used.	The factor analysis produced five dimensions of spatial behaviour, which are as follows: the significance of the residential neighbourhood when it comes to spaces for activity; the number of activities available; the size, eccentricity, and specialisation of the activity space; and finally, the importance of the residential neighbourhood in the activity space	The primary weakness of the study is that the data on frequent mobility were self-reported, and the researchers did not take environmental factors into account
	[67]	In the metropolitan region of Helsinki, Finland, there are two distinct age groups of adults: younger and older	The findings indicate that there is a large divide between the two groups with regard to the frequency as well as the associated factors of a variety of multi-local travel habits	We were able to examine the impacts of many factors with support from statistical tools in this study, but without longitudinal data, causality of relationships cannot be clearly verified. A frequent disadvantage of all data acquired using map-based surveys is that there may have been some biases added to the study due to variances in the amount of participation in mapping among participants and disparities in their level of mapping abilities. They were obtained separately and not meant for comparison, even though they were collected using the same approach and through a comparable survey
	[68]	It is hoped that this study will shed light on how seniors cope with their socio-spatial environment by including both quantitative and qualitative approaches, as well as spatial analytic techniques, into a single study	By adjusting their lifestyle on a regular basis, senior suburbanites implicitly opt to remain in their homes as they get older. Because of their strong sense of self-sufficiency and loyalty to the “suburban way of life”, these people have high housing expectations	
	[69]	A multi-level linear model of geriatric walkability, built environment, and subjective perception is developed in this work in order to illustrate the type and degree of interaction between variables at all levels	The findings reveal that older people’s walking efficiency is influenced by their perceptions of safety and mobility. Either consistency or variation can be shown in the effect on senior walking distance efficiency of built environment variables	
	[70]	Seven hundred twenty-eight Mexican American men and women aged 75 and older were surveyed as part of this cross-sectional research project. Multiple regressions were used	The lives of older Mexican Americans in the United States were severely constrained, with nearly 80% of them confined to their homes or immediate neighbourhoods. There was a correlation between having less life space and older age, female sex, stroke, high levels of depressive symptoms, BMI of 35 or above, and ADL difficulty.	It will be necessary for future research to investigate the correlation between life space and health outcomes, as well as to characterise the development of life space over the course of time in this group.
	[71]	Using data from the 2011 Hong Kong Travel Characteristics Survey (TCS), we identified three distinct categories of out-of-town travel: required, maintenance, and discretionary. There were two models employed: Poisson and linear regression	During particular times of the day, certain older adults with specific socioeconomic and geographic characteristics confront possible geographical hurdles to meeting their mobility demands	Were not described
Opportunities	[72]	Real-life GPS data from 95 older persons were used to develop a collection of mobility indicators that reflects various elements of everyday movement	There are several studies that do not adequately reflect the time, distribution, and stop-move segmentation of motions. To provide a complete picture of an older person’s everyday movement, factor analysis uncovers the following six dimensions: living space, the number of activities performed outside of the house, the amount of time spent using active transportation modes, the stability and extension of living space, and the timing of mobility	Poor data quality due to low participant compliance or particular movement patterns (staying a lot indoors) that are linked to low socioeconomic level. For healthy ageing outcomes, it is not necessary to identify which aspects of mobility are most significant (e.g., active living, independence, social participation)
	[73]	Patterns and motifs are used to help us. Various daily movement patterns were associated with socio-economic and built-environment variables	About 82% and 86% of all senior replies on workdays and non-workdays, respectively, may be summed up in 15 different themes. When it comes to non-workdays, seniors are more likely to display basic motifs with three or fewer unique places, but during workdays, they tend to display more complex patterns	No strong generalizability can be derived from research on senior daily mobility patterns and the relationship between these various patterns of daily mobility and socio-demographic indicators as well as built environment components This study exclusively examines the travel habits of those above the age of 60. Our results are compared to other studies, however a direct comparison between persons over 60 and those under 60 is not possible. To get a fuller picture of how people engage inside their own households, a more in-depth look at how they interact with each other within their own households is required
	[74]	Drawing from the findings acquired from time–space diaries and surveys filled by older adults in three Czech cities, the model of socio-spatial isolation was established	That social exclusion is a multifaceted (comprising of passiveness, isolation, and loneliness) issue that is both geographically specific and gendered is suggested by the research. A person’s level of passivity is closely linked to their income and their participation in recreational sports. Age, gender, and educational attainment, as well as the regularity with which a person works and engages in certain leisure activities, all of which are limited by health issues, financial resources, and physical mobility, all contribute to a sense of isolation	Sample size is small, and there are fewer variables in the model as a result (e.g., interaction terms are limited to the socio-demographic)
	[52]	Elicitation of important knowledge using a systematic approach. The scenario model is implemented and validated using the OBO Edit tool	Using a scenario model, the first step in providing help to people with disabilities who are able to move around outside is being taken. Building an ATD capable of providing situational awareness aid and perhaps improving the quality of life for folks presenting with disabilities is based on this approach	Despite the study’s limitations, the use of GPS as the next best available practical approach was adequate as a reference standard to determine LSM.
	[52]	The LSA-CI was used in a cross-sectional study of 118 multimorbid older adults with cognitive impairment to document their life-space mobility (LSM). The study’s findings were supported by GPS-based distance measurements to the researcher’s house.	Clinical treatments can be tailored to the LSA-CI cut-off score and monitored over time	Limited analysed factors
	[75]	The study was conducted using a longitudinal cohort design (*n* = 33) with older persons who resided in the community. At baseline and one year later, GPS data and activity records were obtained	When paired with qualitative data, GPS technology is a powerful and valuable tool for acquiring new information. The use of GPS recorders in assessment and intervention design is an option	GPS and movement logs were only gathered for two periods of time. As the sample comprised mostly Caucasian females with access to personal automobiles, the categories described by these individuals cannot be generalised to other populations. Elders who indicated high levels of self-perceived well-being were included in the study
	[33]	Data gathered by employing GPS receivers in conjunction with questionnaires and time diaries	A new interpretation of the Ecological Press Model of Aging was created using the findings, which incorporated family structure as a moderator of the effects of environmental stress. In the long-standing interaction between the environment and the health of older persons, social constructions play a critical role.	This study had a limited sample size of 30 people, thus the findings should be used as a starting point for further research in the field of ageing.
	[76]	This is modelled by differences in demand intensity and mobility	Seniors have a greater demand severity and are less mobile than their non-elderly counterparts. More healthcare funds should be provided and better public transit to institutions, particularly in rural regions, is needed to increase the availability of healthcare for the older adults	An older person’s healthcare use behaviour is modelled using only two variables (will be solved in future research). Second and third-tier hospitals are handled equally in this study (next research will examine if a hierarchical pattern exists among these institutions). The older population’s demand is assumed to be the same throughout the research region
	[77]	A mixed-method approach was used in the research, which included both qualitative and quantitative components. A total of nine German cities with various spatial structural features took part in the study (140 qualitative interviews and 5500 surveys)	The results show a strong affinity to the area in which they live and the people who live there. There is a good chance that future older persons will be able to stay in their homes for a long time because of the large percentage of people who own their own homes. The few people who plan on moving within the region or using their second homes more regularly are likely to live in various locations in the future	There are not many outcomes. Geographically, a more in-depth worldwide comparison of persistence would be a worthwhile study project in the future
	[40]	In Beijing, China, a survey of 139 participants (i.e., older individuals aged 60 and above with varying degrees of disability) was conducted in three separate areas	(1) In order to effectively assist disabled older people to age in place, we characterised unmet needs for older adults care (ADL and IADL assistance) among community-dwelling disabled older adults; (2) discovered disabled older adults had much more unfulfilled necessities for both ADL and IADL aid due to a lack in connections to care resources; and (3) characterised the linkages to care assets for stronger support disabled older adults to live independently.	There is a problem with the sample size. Self-reporting or surrogate reporting was used to obtain all data. As a result, the variables may be overestimated or underestimated
	[78]	There were 844 participants in the study who were 55 years or older and lived in the Helsinki Metropolitan Area in Finland. They completed a map of their daily errands and indicated which mode of transportation they used and how often. Participants’ walking excursions were culled from the data, and the impacts of personal, psychological, and environmental factors on older individuals’ ability to walk were studied both directly and indirectly	There was a strong correlation between the number of older adults who walked to work and where they lived, as well as the number of crosswalks, transit stops, and recreational sports venues in the area. Walking by older individuals was shown to be most affected by the number of transit stops within walking distance. Individuals’ demographic and psychological characteristics had little bearing on whether or not the built environment affected their ability to walk as they aged. Walking was positively influenced by a person’s level of education and fitness aspirations, but wealth had a negative impact. Physical activity objectives were an indirect indicator of how gender and self-perceived health affected walking	It is possible that the PPGIS approach as a whole limits the investigated population group since people who have inadequate computer literacy or no internet access are not included in the study measurement. Detailed information on destination density would have been useful, however this was not attainable due to a lack of data
	[79]	Observational and questionnaire surveys, 687 seniors aged sixty-five and older	A person’s biology is not the most essential component. Instead, even the oldest-old group of eighty-five and older can be more active if they maintain a healthy weight range and utilise a walking assistance properly	Because of the lack of local resident registrations, the samples were not selected by random sampling
	[80]	Accumulative behavioural scenario variants, Albatross system as the simulator	The older adults of the future will have to work longer hours, adjust their activity pattern to include more social and leisure activities, escape the morning rush hour by changing their schedules, and maybe add more spatial variety into their living arrangements	not described
	[81]	A community-based survey (Philadelphia, 2010) assessed mobility (Life-Space Assessment [LSA]; range = 0–104) of older adults (*n* = 675, census tracts = 256). Social capital was assessed for all adults interviewed from 2002–2010	Mobility may not be as influenced by social capital as other local variables. Research on mobility should take into account the interplay between neighbourhood and individual factors	There may have been a reduction in the measure’s capacity to identify variations in movement between groups as a result of replies being standardised
	[82]	Forty-four participants who took part in a reality cave exercise and a sub-group of 10 people who visited an unfamiliar area as pedestrians describe their experience of walking a predetermined route.	In unfamiliar situations, there are a variety of issues that older people face, such as bad signage and confusing places as well as poor pavement and “sensory overload”, which refers to the noise and complexity of the surroundings. Participants relied more on landmarks and unique structures to help them find their way through new locations	Low sample size
	[83]	92 global positioning system (GPS)-recorded mobility tracks from 47 participants (24 women, 23 men) from the Cardiovascular Health effects of Air pollution in Telangana, India (CHAI) project (mean: 4.1 days/person). The mean age was 44 (standard deviation: 14) years	Using a principal component analysis, it was discovered that 86% of the variance in women’s and 61% of the variance in men’s mobility can be attributed to the size of the activity area, the mobility of the dwelling and the mobility within the hamlet. All three characteristics were linked to a person’s age, socioeconomic position, and level of urbanisation	As a result, it is difficult to examine how unique person traits affect the correlation between predictors and mobility indicators. However, our automated system was unable to discriminate between time spent indoors and outside. data gathering may not be reflective of a person’s mobility patterns if it takes place in a short period of time
	[84]	Using data gathered through in-depth interviews, follow-along interviews, and mapping activities, three individuals’ perspectives on ageing at home in Dublin are explored. Participants’ place-based functionings are shown through the use of annotated maps	There are many diverse ways in which people value and negotiate their place-based functioning, relying on their requirements, preferences, and health or mobility issues. Additionally, the results show the significance of helpful settings and social assistance in facilitating older adult individuals to accomplish their greatest valued functioning’s over time, such as being able to get outside, start engaging and communicate with everyone, complete daily duties and odd jobs assertively, and continue to stay self-sufficient	It is possible that subsequent study may examine the ageing experience of people from varied socioeconomic and ethnic origins, including those who are homebound, in a broader range of circumstances. Additionally, these strategies might be developed over time
	[85]	Participants in focus groups	According to the resultant walkability typology, most urban street blocks offer only modest protection for the older adults. Older adults who dwell in seniors’ apartments may be compelled to engage in indoor activities rather than participate in urban life, resulting in a sort of spatial isolation	Limited sample size
	[86]	49 older adults from the Tel-Aviv Metropolitan area (Israel). Participants were tracked for an average of 28 consecutive days using a location kit that combined GPS with RFID technology	Demographics, sex, and ease of access to a private vehicle all play a crucial role in determining how far senior persons with cognitive impairment can travel outside the home. These patterns show that out-of-home mobility is generally declining	External elements have to be pared down. Additional factors, such as the weather and the length of the day, can be incorporated into the study to determine whether seasonal trends can be discerned
COVID aspects	[87]	GPS-based mobility data for a wider time frame of six months (March 20–August 20) divided into four tiers and analysis for all the US counties (*N* = 3142)	All three mobility matrices (trip rate, out-of-county travel rate, and miles/person travelled) fell until the first wave reached its apex, after which they began to climb. Within- and outside-county travel were found to be inversely associated with infection rates in spatial models. Areas with high concentrations of COVID-infected persons had a higher proportion of those who worked from home, lower percentages of the older adults and educated, as well as a higher proportion of the poor	Because of the nationwide trend of COVID cases, the research duration was partitioned into four tiers, which was not possible to accommodate individually for states with various infection rates and varying timetables for stay-at-home orders and reopening. It is impossible to conclude that each tier of states had a uniform pattern of mobility. It was fair to reduce the amount of granularity in exchange for the study’s relatively long time period, which was divided into four equal halves. Because the study relies on GPS-enabled smartphone data, it excludes persons who do not own a smartphone from the findings
	[88]	Participant responses were mapped out using a map-based questionnaire, and 75–85-year-olds indicated locations for physical activity, places that made it easier to get around outside, and destinations for other activities	During COVID-19, there was a noticeable decrease in activity destinations recorded, with most of those being for physical activity, and they were all located closer to home	In COVID-19, individuals dropped participation due to adverse selection (improved health and functioning and more competency with digital devices), which reduced the sample size. So the results of this study should be considered with caution and could only be applicable to those over the age of 75 who are well-functioning and technologically proficient. To improve the likelihood of completing the self-completed MQ successfully, sufficient test and training are required. Because all of the variables in the research were self-reported, there is a risk of bias in the data

The issue of mobility among older adults can be divided into three thematic units, although due to some overlaps, there are three thematic units based on four clusters. The titles of clusters represent the thematic focus, with subtitles specifying the main topic within each cluster. The first group, termed ‘challenges’, primarily based on cluster 1, encompasses accessibility, personal ability and disability, and factors related to older adults’ behaviour, QoL, and health complications that are relevant to mobility. This area focuses on personal challenges, self-activity, healthy lifestyle, rehabilitation, and exercise that can promote mobility. The second area, termed ‘conditions’, based on clusters 2 and 4, focuses on defining individual and environmental conditions that affect mobility and accessibility. The last area is related to community, facility, technologies, and individual possibilities. It explores opportunities to improve mobility, treatment models in healthcare, the use of modern technologies to promote mobility, and new technologies for its monitoring and research. This topic also considers research on facilities where older adults receive care, as these often incorporate equipment with modern technologies, as indicated by cluster 3, particularly evident form Scopus keywords in Table 6. The individual topic focuses on the interplay between neighbourhood and individual factors, such as health conditions and individual perception of mobility as an opportunity to influence overall health status, improve metabolism, overcome some disabilities, and reduce the risk of mortality. Table 6 summarises 52 studies considered crucial within the above thematic units.

Regarding the determinants of mobility, several main findings have been identified. The selected determinants for further consideration are those that appear in multiple studies.

The total number of trips, including trips away from home, tends to decrease among older adults in higher age groups. Reduced mobility in people over the age of 65 is associated with factors such as living alone, physical disabilities, reliance on foot or bicycle transportation, lower education levels, longer distance to public transportation, inability to drive, and residing in less affluent areas. Furthermore, the frequency of work-related and shopping trips decreases with increasing age. Older adults who live alone travel less for work and leisure activities. Individuals with severe disabilities make fewer trips for work, shopping, and recreation, but they have more frequent doctor visits. The presence of employment influences travel patterns, with employed individuals making more work-related trips and fewer trips for physical activity, recreation, and socialising. The frequency of their outings for shopping, physical activity, leisure, and socialising remains consistent on weekdays and weekends. Gender primarily affects the frequency of shopping trips. Older adults who are less likely to travel outside their immediate neighbourhood tend to be those who are more elderly, severely disabled, rely on the bicycle, walking, or local bus as their primary mode of transportation; are less educated; live further than 10 min from the nearest subway; or are unable to drive themselves. Travelling for pleasure is less common, but activities such as shopping, dining, attending church, and seeing friends are more frequent among those aged 65 to 69 years old (i.e., the youngest older persons). This age group engages in a variety of activities, often within their neighbourhoods. In terms of the frequency of leisure trips, older adults aged 80 or more years come in second, followed by those who are severely incapacitated [64].

The mobility habits of older individuals are influenced by the time of day and the location in which they reside. Mandatory journeys tend to be shorter, while non-mandatory excursions are longer, especially during off-peak hours when transportation options are more readily available. Although their impacts are less pronounced than previously suggested, they nonetheless have a major impact. Age-specific light buses, which have evolved from a legislative concept to community-based organisations, offer transportation services for older persons within their districts [71].

In 2017–2018, older individuals reported frequent visits to various activity sites, including museums, libraries, and parks. Exercise, mobility, and other activities linked to everyday life (e.g., grocery shopping and the utilisation of health and meal services) were all included in these destinations of cultural and social visits. COVID-19 restrictions enforced by the Finnish government in 2020 resulted in older individuals reporting only physical exercise-related destinations as their preferred mode of transportation. As a result of these restrictions, it was either difficult or unpleasant to participate in other activities (e.g., eateries, museums, and planned collective events). During the COVID-19 pandemic, additional activities of individuals were primarily tied to their everyday routines. According to previous research, older individuals were more physically active when they were near their homes, and the elderly spent more time at home and within their neighbourhoods when mobility restrictions were in place to minimise the spread of COVID-19 [88, 89].

Based on the current state of knowledge, especially from the areas of strategic documents and government acts, it can be assumed that availability, local mobility systems (LMS), and technologies are important for the mobility of seniors. In the following text, the findings from the scoping review are therefore interpreted in relation to these assumptions.

#### Zones of living space

In the review process shown in Fig. 5, the findings of the studies were classified into three groups, with one group named ‘condition’ and further divided into individual and environmental subgroups. These classifications were connected to five zones of living. As shown in Table 6, the studies focus mainly on the external environment, such as neighbourhoods and distant places.

Table 7 shows the distribution of papers, indicating better coverage for zones 4–5 [90], while papers focusing on in-house mobility are less frequent in the sample. Regarding environmental conditions, studies indicate determinants that affect senior mobility. For instance, Peel et al. [91] found that living space was related to mobility, physical performance assessments, transportation problems, mental state, and depression in a population-based sample of seniors.

**Table 7:** Clusters and their subgroups in relation to individual and environmental conditions

**Title**	**Zone 1**	**Zone 2**	**Zone 3**	**Zone 4**	**Zone 5**
	Other rooms than the bedroom	Area outside the house	Neighbourhood	Outside neighbourhood, but in town	Outside town
**Individual condition** (age, home, gender, care, family, travel behavior, mobility, migration, risk factor)					
[55]	**x**	**x**			
[60]	**x**	**x**	**x**	**x**	**x**
[35]	**x**	**x**	**x**	**x**	**x**
[66]	**x**	**x**	**x**	**x**	**x**
[68]				**x**	**x**
**Environmental condition** (residential mobility, distance, neighbourhood, transportation, urban area, residence characteristics, socioeconomic factors, urban population, spatial mobility, place, context)					
[54]				**x**	**x**
[56]		**x**	**x**	**x**	**x**
[57]				**x**	**x**
[58]	x	x	**x**	**x**	**x**
[59]	x	x	x	x	x
[61]	x	x	x	x	x
[41]	x	x	x	x	x
[62]				x	x
[63]		x	x	x	x
[34]	x	x	x	x	x
[64]		x	x	x	x
[65]			x	x	x
[67]			x	x	x
[69]					
[70]	x	x	x	x	x
[71]				x	x

Different zones of life space were associated with performance-based measures of function, namely, visual impairment, and several measures of lower extremity motor function. Moreover, even after controlling for demographic variables (age, sex, education, and time of year), visual impairment, and lower extremity motor function, life space was also associated with levels of global cognition, extraversion, and having a future goal-oriented purpose in life. Higher scores on each of these variables were associated with larger life spaces.

A decline in LSM is associated with a decline in QoL among community-dwelling older people, even after considering potential confounders. The association between the decline in LSM and QoL can be explained in several ways. First, diminished opportunities for participation in outdoor activities and the resulting insufficient social interaction may lead to social isolation and loneliness, which, in turn, may lead to poor QoL [92]. Second, reduced time spent outdoors and hence, lower levels of physical activity and increased sedentary behaviour may have adverse effects on health and thus a negative influence on QoL [93]. Spending time outdoors may also have a direct effect on QoL, as people, especially in the Finnish context, often like to enjoy the outdoors, which has been shown to enhance their well-being [32]. Third, a decline in LSM may indicate difficulties in taking care of daily errands independently, which may also contribute to a sense of losing control over one’s life [61].

Increased LSM is associated with objectively measured physical activity indicators, such as step count, moderate and low activity time, and sedentary time. Going outdoors in the neighbourhood at least once a week has been found to be beneficial for maintaining physical function in frail older people. Finding ways to encourage community-dwelling older people to go outdoors more often may increase their physical activity level and help to maintain their physical function. Poor health, low physical activity, and mobility limitations often coincide in the same individuals [34].

Some studies explore the link between mobility and other aspects of the natural environment. To give one example, Hinrichs et al. [62] have discovered that elderly people who perceive parks or green spaces in their neighbourhood as encouraging outdoor mobility are more likely to visit their regular grocery shop, regardless of whether they walk to the grocery store or not. The impression of a path as a mobility facilitator is not correlated with an increase in the likelihood of walking for transportation. Additional factors positively associated with walking as a mode of transportation include low private car use, short distance to the store, and a high level of street connectedness.

#### Accessibility

In terms of accessibility, barriers that affect seniors are identified in their immediate vicinity, including the condition of the terrain and the availability of services, doctors, and basic facilities. The field of research in this area is rapidly developing, with preparations being made to support governments and regions. According to the findings of Ståhl et al. [94], older individuals face mobility issues related to accessing and navigating bus stops, as well as getting on and off buses. Transitioning from a fixed-route system that is not accessible to one that is fully accessible, including the cars, stops, and stations, may take many years. The structure of public transportation networks is determined by the demographics of the population. Rural and suburban communities, particularly those with low population density, still face challenges in terms of mobility. Older individuals living in rural settings are more likely to experience social isolation and have limited socialisation options apart from their families and friends compared to their suburban counterparts [95]. According to the results of Siren et al. [96], providing older people with general mobility and accessibility is essential for their independent living, but it is equally important to support activities that enhance their sense of community belonging. 

Physical determinants of mobility within the neighbourhood can include unsuitable environments and equipment at stops, difficulties in boarding and alighting, suboptimal air or temperature conditions inside, orientation in timetables, lack of shade, greenery, toilets, railings, benches, lighting, views of the surroundings, obstacles such as stairs, curbs, parked cars, bicycles, other obstructing objects (posts, stands, bushes), noise, and unpleasant environments. Older adults living in rapidly urbanising environments face challenges in either integrating into or becoming isolated from the society [97]. The concept of New Urbanism and Transit-Oriented Development emphasizes compact forms, mixed-use, high density, and convenient public transportation [98] in order to facilitate spatial mobility among the ageing community and ensure productive ageing. 

#### Mobility aspects under consequences of development emerging technologies

The study of topics and keywords in the current period of spatial mobility research has revealed that technological factors are playing an increasingly significant role. Scholars have recognized that the spatial mobility of older adults cannot overlook the technological dimension, which has the potential to significantly improve their physical and mental capabilities and performance. However, there is also a concern about an increase in social inequity due to the affordability and accessibility of such technological tools. Technologies are being developed to focus on mobility within the home, as well as on mobility and safety outside the house and in the neighbourhood. New technology is being designed to address geriatric mobility problems such as falls, bed rail entrapment, patient management, and wandering. These technologies aim to prevent or reduce adverse events that hinder therapy, delay rehabilitation, exacerbate impairment, and jeopardize patient safety. Examples of such technologies include hip guards, wheelchair and scooter safety systems, smart walkers, falling alerts, and environment assistance, which can help to reduce falls and fall-related injuries [99].

Technological solutions for the healthcare needs of the elderly include (1) linking and sharing medical data in networks among users, including medical check-ups, treatment, and care records; (2) providing remote medical care assistance; and (3) utilising AI and robots in treatment facilities to support autonomy [29]. Wearable transfer aid robots are a priority in order to alleviate the physical burden on caregivers. While the usefulness of these robots in nursing homes is still uncertain, they have shown to be beneficial in experimental settings for a variety of activities. Evaluation of care tasks and time ratios while using these robots indicates that they were prominently used for direct patient care in more than 70% of transits, particularly in transfer assistance and toileting [100]. The use of new outdoor and indoor mobility technologies for individual or medical purposes has both advantages and disadvantages, as shown in Table 8.

**Table 8:** Tools supporting mobility and related barriers

	**Indoor**	
	**Opportunity**	**Barriers**
For medical specialist care	• medical check-up records and treatment and care records • remote medical care practice services • use of new technologies (AI, robots) which bring improved patient engagement • cost savings by reducing the need for manual labour and increasing efficiency • more effective with other healthcare providers • better access to data	• protection of personal data • time to learn and implement new technology into their workflows • doctors prefer to stick to traditional methods of patient care • the cost of purchasing and implementing new technology • different systems and devices may not be able to communicate with one another
For individual use	• improved health outcomes by use: hip protectors, wheelchair/scooter safety features, intelligent walkers, fall alarms, environmental aids, robots, transfer assistance and toileting care • increased access to information • improved communication with healthcare providers • better tracking of personal health data • more convenient and accessible care • more active role in one’s own care	• fear or distrust of technology • a lack of experience with touchscreens and other user interfaces • health conditions, such as vision and hearing loss, may make it difficult • high cost of smart technology, medical devices
	**Outdoor**	
	**Opportunity**	**Barriers**
For medical specialist care	• improved patient outcomes • remote monitoring • increased access to information • more informed decisions about treatment and care	• unsuitable environment and equipment of stops • low frequency of connections • difficulties in boarding and alighting
For individual use	• improved health outcomes (by using wearable devices and other technology, patients can better track their health, including their physical activity, heart rate, and other vital signs) • increased access to information, and more convenient and accessible care • improved safety (by use of fall detectors, voice-activated assistants, medication reminders)	• cost of purchasing and implementing new technology • problem with conditions such as bad air or temperature, orientation in timetables, noise or unpleasant environment, physical barriers in connection with location of toilets, railings, lighting, etc • limited connectivity • battery life • some wearable devices and technology may not be durable enough to withstand outdoor conditions and may break or malfunction easily

To address the challenges mentioned earlier, it is important to ensure that new technology is affordable, easy to use, and secure, and that it seamlessly integrates with existing systems and devices. Furthermore, efforts should be made to provide doctors with training and support to help them to effectively use and implement new technology in outdoor settings. The use of smart technology by seniors presents numerous opportunities for medical specialists, including improved patient outcomes, remote monitoring, increased access to information, improved communication, and better tracking of patient data. To fully realize these benefits, it is important to ensure the seamless integration of new technology with existing systems and devices and to provide seniors with education and support to help them to effectively use and implement new technology in their daily lives.

## Discussion

The objective of the study is to identify the factors that have had a significant impact on mobility in recent years and currently, as well as to identify gaps in our understanding of these factors. The study aims to emphasize areas where further research is needed and where new and effective solutions are required.

Within the scoping review, we employed clustering for database results, which provides an independent view of the structure of the scientific discourse on the given problem. Furthermore, it allowed for the identification of common groups of problems and a neutral evaluation of co-occurrences and associations, not influenced by the researchers’ biases. Several studies from our review report that lower mobility among older people is related to family status, health status, and housing location. Family status plays a crucial role, with factors such as living alone or with someone influencing the motivation and need to run a larger household and to engage in mobility-related activities, such as making trips and visiting family members. If this condition of motivation is met and options are available, for example in the form of a driving licence, health status becomes an important factor. Health status is also significant, as deteriorating mental or physical health tends to decrease the distances travelled by older adults. Within urban areas, concerns about personal safety when using certain means of transportation, such as bicycles or cars, are raised. Several innovative solutions are available to address these issues, as these new solutions have been rapidly developing in the past decade and are being discussed within the context of the Society 5.0 phenomenon.

Webber et al. [16] have established fundamental categories of determinants for older adults’ mobility: cognitive, psychosocial, physical, environmental, and financial categories. The clusters generated from the co-occurrence of keywords in this study cover only a part of these fundamental categories, as specific keywords related to individual financial context or cognitive factors were not significantly investigated in the mobility-related papers. Among the fundamental categories, environmental determinants have been frequently studied, and the ‘condition’ cluster in this study combines environmental and individual conditions. The individual condition cluster is closely related to psychosocial factors, among which Webber et al. [16] include self-efficacy, depression, coping mechanisms, anxiety, fearfulness, and relationships with others, which affect interest and motivation for mobility. The combination of environmental and individual conditions confirms the close interdependence of these factors, as a person’s skill level combined with their surroundings contribute to successful mobility outcomes [101]. Physical determinants are mainly reflected in the ‘person disability’ group of ‘challenges’, indicating that the interpretation of these factors is mainly negative and focuses on the gradual erosion of older adults’ physical abilities related to spatial mobility [16]. Table 9 provides a summary of the main mobility factors discussed.

**Table 9:** Overview of mobility determinants

Source	Categories of older adults’ mobility determinants
[36, 45–47, 52, 60, 86]	Cognitive
[33, 40, 49, 50, 53, 74, 75, 78, 80, 83]	Psychosocial
[34, 35, 40, 54–56, 58, 63, 64, 70, 79, 87, 88]	Physical
[34, 41, 46, 54–57, 59, 61, 62, 64–68, 71–74, 76–78, 81, 82, 85, 97]	Environmental
[54, 71, 73, 74, 83]	Financial – with gender, culture, and biography

The selected studies highlight accessibility discrepancies between seniors and non-seniors, urban seniors and suburban seniors, and seniors who own vehicles and those who do not (e.g. [50]). In older individuals, possessing a valid driver’s licence is associated with a reduced risk of reporting life space constriction and an increased likelihood of life space recovery if constriction occurs. A total of 71 studies demonstrate that older adults have a higher demand for public transit and experience greater mobility disadvantages than younger adults because they are more reliant on public transit. To facilitate access to healthcare for older individuals, additional healthcare resources should be committed and public transit to healthcare facilities should be improved, particularly in outlying areas. In a total of 72 studies, the results indicate a strong attachment to the place of residence and the surrounding community among older individuals. The particularly high rates of homeownership suggest that future older adults will continue to live at home. The remaining individuals who are potentially mobile aim to relocate within the region or utilize their second homes more frequently, indicating a likelihood of living in multiple locations in the future.

Healthcare solutions for older adults include connecting and sharing information in a network between users of medical data, including medical check-up records and treatment and care records, providing remote medical care services, and integrating AI and robots in care facilities to promote community independence. GPS technology has proven to be a robust and valuable method for acquiring new information, particularly when paired with qualitative data, as is demonstrated in [72]. GPS data loggers can be incorporated into evaluations and interventions. Rantakokko et al. [61] suggest in their findings that longer GPS use is significantly associated with higher cognitive ability. These results indicate that GPS watches may be able to continuously monitor changes in functional health, allowing for informed preventative efforts.

**Fig. 6:**
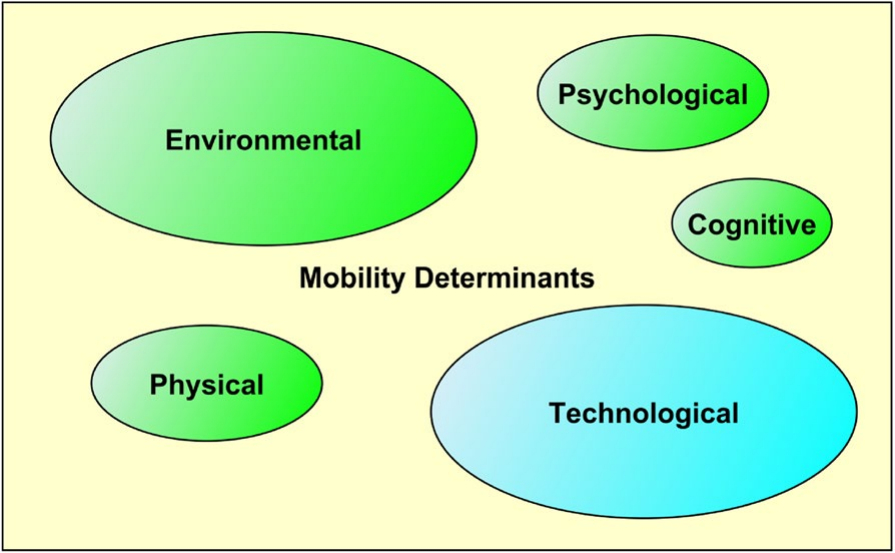
Mobility determinants overview

The determinants of mobility examined in the studies include environmental, physical, psychosocial, and cognitive factors, which are investigated in almost all studies. Financial determinants, along with gender, culture, and biography, are less frequently studied. Additionally, the role of technology and the solutions mentioned in Chapter 4.2.3 on Society 5.0 should also be considered as new determinants of mobility. Figure 6 provides a visual representation of the confirmed determinants of mobility (green), those currently deemed non-essential (yellow), and those that require reclassification (blue).

Johnson et al. [7] have found the following main factors influencing mobility (LSM as an outcome variable): age, gender, education, health issues, and physical capabilities and activities. Our findings confirm the main role of age, education, family status, health issues, and physical capabilities. However, the role of gender as well as biography, culture, and financial aspects has not been extensively studied in the studies included in our review. Johnson et al. [7] provide detailed insight into health issues that contribute to decreased LSM, such as stroke, depressive symptoms, undernutrition, and obesity. Among other factors described by the same authors in their review, the role of car utilization, both as drivers and passengers, is also confirmed, usually combined with walking difficulties. 

In our study, environmental conditions and locality play an essential role in mobility. Johnson et al. [7] identified a single paper oriented at environmental factors related to restricted LSM (Rantakokko et al. [61]). This particular paper reported environmental barriers, such as high curbs, lack of sidewalks, and missing environmental facilitators, including safe crossings or good lighting. Our findings substantially extend the list of environmental factors and underline the roles of other factors, such as unsuitable environment and equipment of stops, difficulties in boarding and alighting, poor air conditions, orientation in timetables, shade, greenery, toilets, railings, benches, views of the surroundings, stairs, parked cars, bicycles, obstructing objects, noise, or unpleasant environment, and non-aesthetic factors. Understanding which environmental factors are perceived as significant and how they can be improved is crucial for enhancing elderly mobility.

The present research has generated valuable discoveries, but it is important to acknowledge its limitations. To begin with, the precision of search keywords may have led to the exclusion of relevant papers from our study, and the selection of keywords influenced the investigated zones of living, so that, for instance, the coverage of in-house mobility is limited.

Second, differing aims, demographics, and findings were used to classify the publications under analysis. We have excluded travelling for recreational purposes to distinguish gerontological and travel studies related to different determinants. Finally, clustering results in some overlap of topics, which are therefore not as clearly delineated as if they were formulated by researchers. However, such overlap allows an insight into how these factors are interlinked and mutually conditioned.

## Conclusion

Reduced mobility among older adults is a complex problem stemming from many areas of human activity and perhaps has more options for improvement. The results of this study reveal that the key determinants of mobility are environmental, physical, cognitive, psychosocial, and technological. In relation to the previous results, we underline the role of modern emerging technologies in overcoming disabilities (or more common geriatric mobility problems). We also emphasise the role of the environment and the interactions between individuals and the environment. Additionally, we have identified more environmental factors than previous studies, including the role of suitable equipment of dwellings and public spaces, as well as street furniture. Based on these key determinants, future research can focus on several areas, including:

- understanding the interactions between different determinants of mobility: Further research could examine how the different determinants of mobility interact with one another and jointly influence mobility outcomes.
- Addressing environmental factors that impact mobility: Research could examine the impact of environmental factors such as air pollution, climate change, and access to green spaces on mobility outcomes and identify interventions to address these factors.
- Identifying interventions to improve cognitive and psychosocial determinants of mobility and examining the impact of mobility on health and well-being.

Given the importance of technological determinants of mobility, future research could focus on developing new technologies or improving existing ones to enhance mobility outcomes. In the context of emerging technologies, important opportunities for future research include:

- integration of smart home technologies to enhance accessibility and safety for seniors in their homes,
- development of assistive technologies for mobility, such as exoskeletons and robotic devices, to help seniors with physical limitations move around more easily,
- design of accessible and age-friendly public spaces, such as sidewalks, public transportation systems, and buildings, to improve mobility for seniors in their communities.
- utilization of AI and machine learning algorithms to optimize transportation systems and make them more accessible to seniors.
- study of the impact of autonomous vehicles on senior mobility and the development of policies and regulations that support their safe and equitable use by seniors.
- investigation of the relationship between physical activity, mobility, and quality of life for seniors and the development of interventions to support healthy ageing.
- exploration of innovative financing models to support the integration of assistive technologies into the lives of seniors, including the use of public-private partnerships.

Further research on these determinants can help arrive at solutions that, in turn, will improve mobility. Financial determinants have not been widely investigated, so future research should focus of financial determinants, especially since many technological solutions are expensive and not commonly available, which limits their use. 

The number of older adults is increasing in developed countries, and there is increased consumer potential. This may be a strong motivation for public and private entities to develop innovative and supportive activities for this target group. Addressing mobility challenges for older adults involves a multidisciplinary approach that includes healthcare providers, policymakers, urban planners, and families.

## Supplementary Information

**Additional file 1: Figure A1.** Sunburst chart on most recent keywords and most cited keyword. **Table A1.** Keywords with the highest Total Link Strength (TLS) to the keyword ‘mobility’ in Web of Science. **Table A2.** Keywords with the highest TLS to the keyword ‘accessibility’ in Web of Science. **Table A3.** Keywords with the highest TLS to the keyword ‘spatial mobility’ in Web of Science. **Table A4.** Keywords with the highest TLS to ‘mobility’ in Scopus. **Table A5.** Keywords with the highest TLS to ‘spatial analysis’ in Scopus.

## Data Availability

The datasets used and/or analysed during the current study available from the corresponding author on reasonable request.
