# Supplementary material for: Challenges and opportunity in mobility among older adults – key determinant identification
Source: BMC Geriatr. 2023 Jul 21;23:447. doi: 10.1186/s12877-023-04106-7 (PMC10360303; doi:10.1186/s12877-023-04106-7)
Supplement: Supplementary file 1 — Additional file 1: Figure A1. Sunburst chart on most recent keywords and most cited keyword. Table A1. Keywords with the highest Total Link Strength (TLS) to the keyword ‘mobility’ in Web of Science. Table A2. Keywords with the highest TLS to the keyword ‘accessibility’ in Web of Science. Table A3. Keywords with the highest TLS to the keyword ‘spatial mobility’ in Web of Science. Table A4. Keywords with the highest TLS to ‘mobility’ in Scopus. Table A5. Keywords with the highest TLS to ‘spatial analysis’ in Scopus. [file 12877_2023_4106_MOESM1_ESM.docx]

**Annex A**


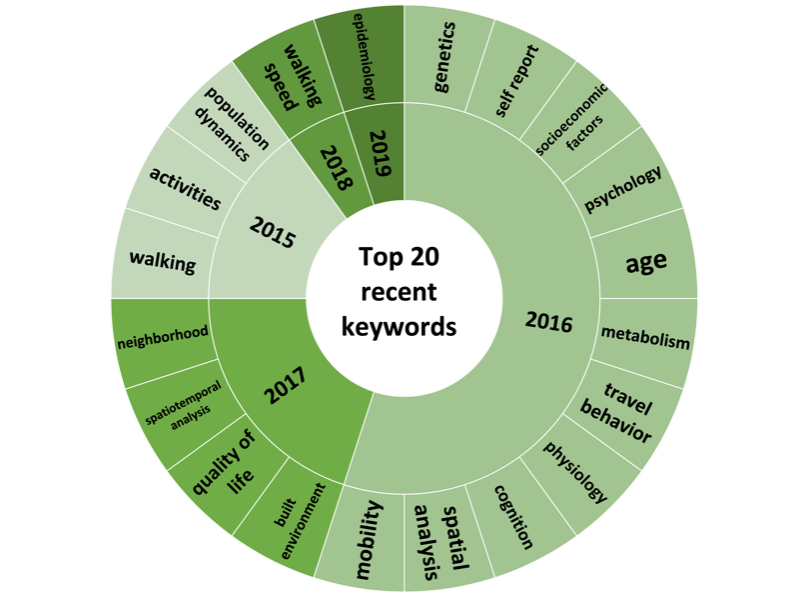

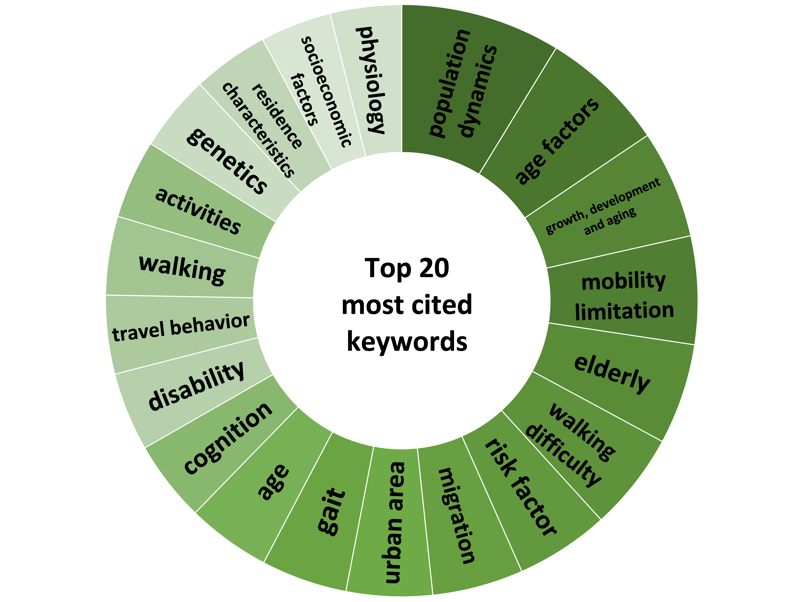


**Figure A1: "**Sunburst chart on most recent keywords and most cited keyword"

VOSviewer created the most frequently utilised and referenced terms that have substantial appearances with the search, in terms of the total number of co-occurrence phrases and the intensity of the co-occurrence relationships (Fig. A1). Sunburst charts show the top 20 recently searched keywords that co-occurred and the top 20 cited keywords that co-occurred with the keywords search. Top recent keywords are ‘epidemiology’, ‘walking speed’, ‘neighborhood’, ‘spatiotemporal analysis’ and ‘quality of life’. While top most cited keywords are ‘population dynamics’ (65 citations), ‘age factors’ (51 citations), ‘mobility limitation’ (44 citations), ‘growth’, ‘development and aging’ (44 citations), and ‘walking difficulty’ (40 citations). These keywords can help scholars understand the trend of the keywords in their search.

| **Table A1.** **Keywords with the highest Total Link Strength (TLS) to the keyword ‘mobility’ in Web of Science** | | |
| --- | --- | --- |
| 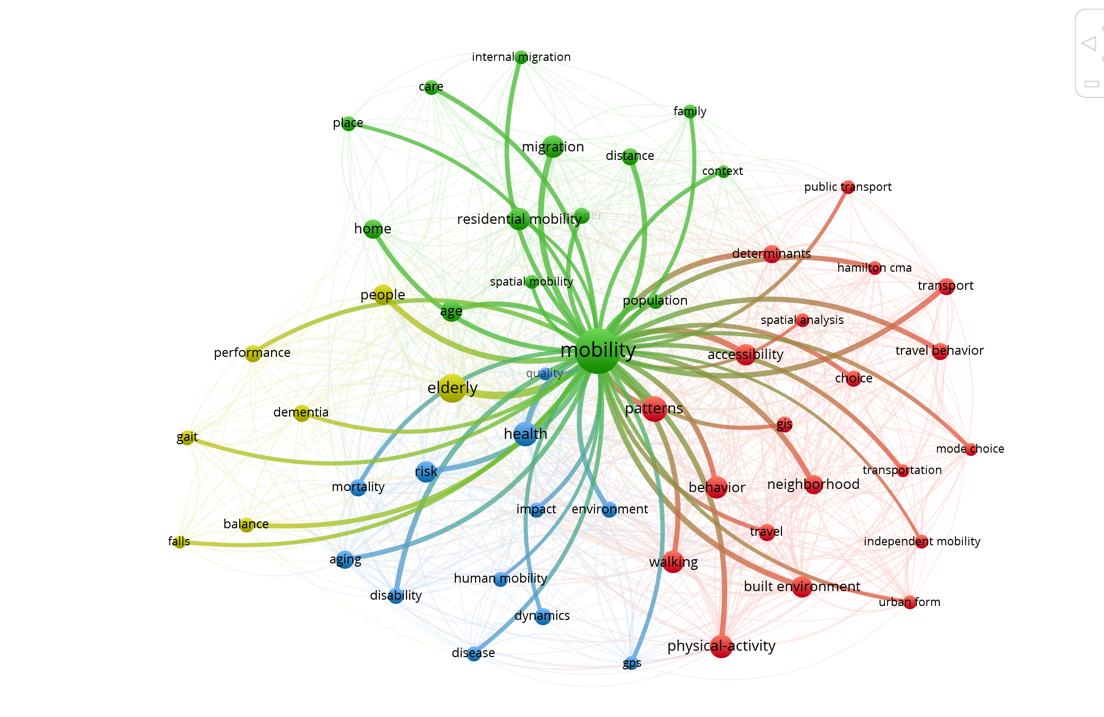 | **keywords** | **TLS** |
|  | patterns | 15 |
|  | health | 14 |
|  | migration | 14 |
|  | built environment | 12 |
|  | transport | 11 |
|  | risk | 10 |
|  | disability | 10 |
|  | neighbourhood | 10 |
|  | physical activity | 10 |
|  | distance | 9 |

Tables A1, A2, and A3 show the 10 ten keywords that are most closely linked to the keywords ‘mobility’, ‘accessibility’, and ‘spatial mobility’ in Web of Science and might become the determinants to the keywords search. For example, ‘patterns’, ‘health’, ‘migration’, ‘built environment’, ‘transport’, ‘risk’, ‘disability’, ‘neighborhood’, ‘physical activity’, and ‘distance’ were linked to ‘mobility’ in WoS.

Concerning ‘accessibility’, two subdomains are formed – transport-related (transport, Hamilton CMA, travel behaviour) and walking/health-related (walking, health, built environment, physical activity), Total Link Strength (TLS) 14 and 12 resp. Searching ‘accessibility’ and ‘elderly’ clearly shows what is important for this connection – walking, usually understood as a way to improve a health status, and transport issues.

The ‘spatial mobility’ graph is vague but indicates where the context of spatial mobility is stronger – home and (close) neighbourhood.

| **Table A2:** Keywords with the highest TLS to the keyword ‘accessibility’ in Web of Science | | |
| --- | --- | --- |
| 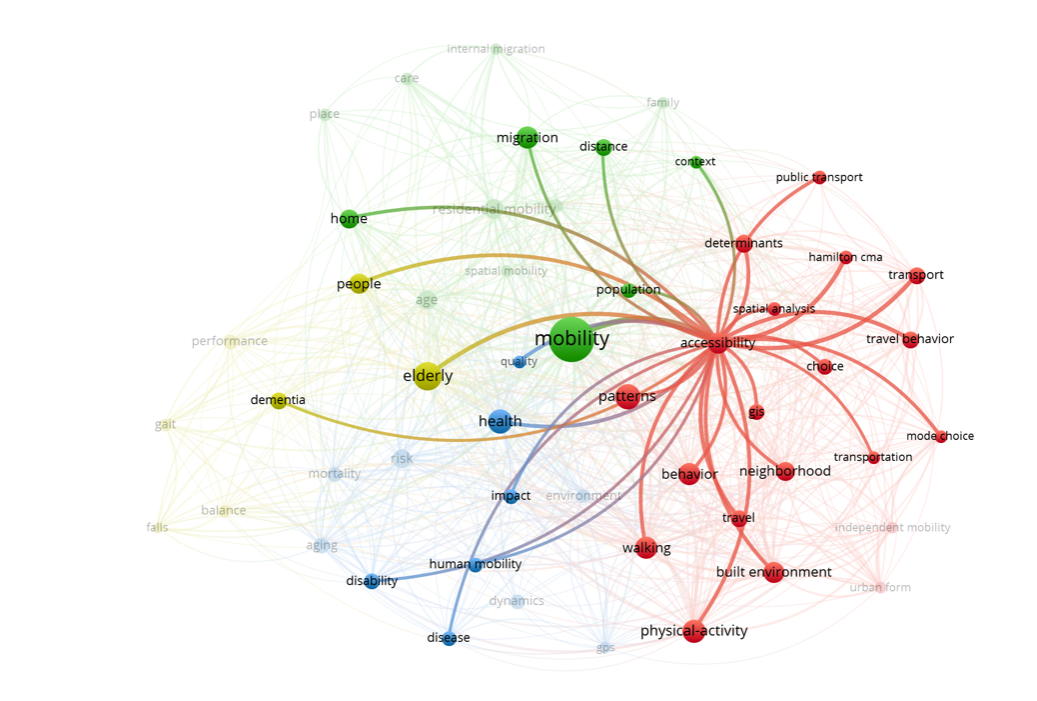 | **Keywords** | **TLS** |
|  | Mobility | 14 |
|  | Transport | 5 |
|  | hamilton CMA | 5 |
|  | travel behavior | 4 |
|  | Quality | 3 |
|  | Walking | 3 |
|  | Health | 3 |
|  | built environment | 3 |
|  | physical activity | 3 |
|  | GIS | 2 |
| **Table A3:** Keywords with the highest TLS to the keyword ‘spatial mobility’ in Web of Science | | |
| 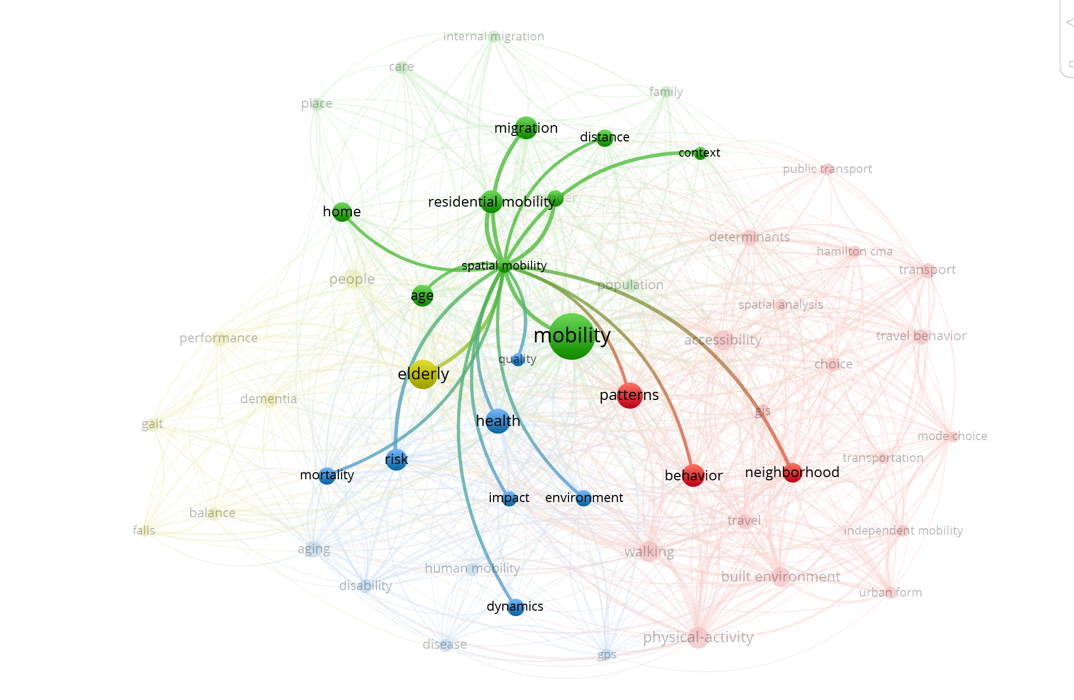 | **Keywords** | **TLS** |
|  | Risk | 4 |
|  | Home | 3 |
|  | Age | 3 |
|  | residential mobility | 3 |
|  | Migration | 2 |
|  | Distance | 2 |
|  | Context | 2 |
|  | Gender | 2 |
|  | Neighborhood | 1 |
|  | Patterns | 1 |

‘Mobility’, ‘transport’, ‘hamilton CMA’, ‘travel behavior’, ‘quality’, ‘walking’, ‘health’, ‘built environment’, ‘physical activity’ and ‘GIS’ were linked to ‘accessibility’. The top 10 keywords that linked together to ‘spatial mobility’ were ‘risk’, ‘home’, ‘age’, ‘residential mobility’, ‘migration’, ‘distance’, ‘context’, ‘gender’, ‘neighborhood’ and ‘patterns’. But, due to the limited number of publication consisting the keyword ‘spatial mobility’, the TLS were lower compared to other keywords. One of the strengths of this study is to find the well-grounded determinants to the keyword ‘spatial mobility’.

| **Table A4:** Keywords with the highest TLS to ‘mobility’ in Scopus | | |
| --- | --- | --- |
| 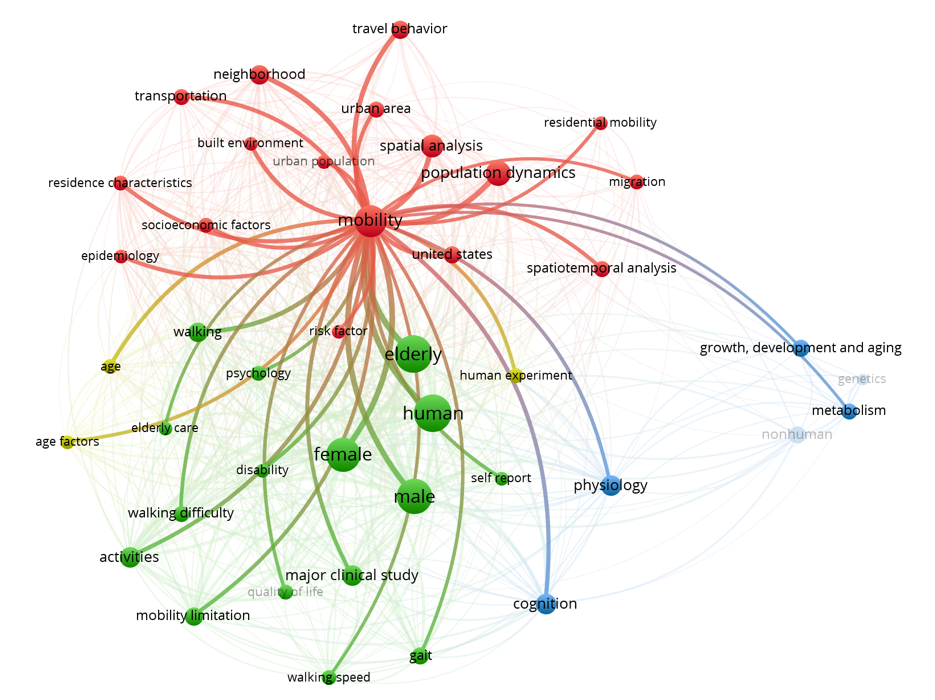 | **Keywords** | **TLS** |
|  | population dynamics | 21 |
|  | spatial analysis | 16 |
|  | neighborhood | 14 |
|  | travel behavior | 13 |
|  | walking | 10 |
|  | built environment | 8 |
|  | residence characteristics | 7 |
|  | activities | 7 |
|  | cognition | 7 |
|  | transportation | 7 |
|  | epidemiology | 6 |
| **Table A5:** Keywords with the highest TLS to ‘spatial analysis’ in Scopus | | |
| 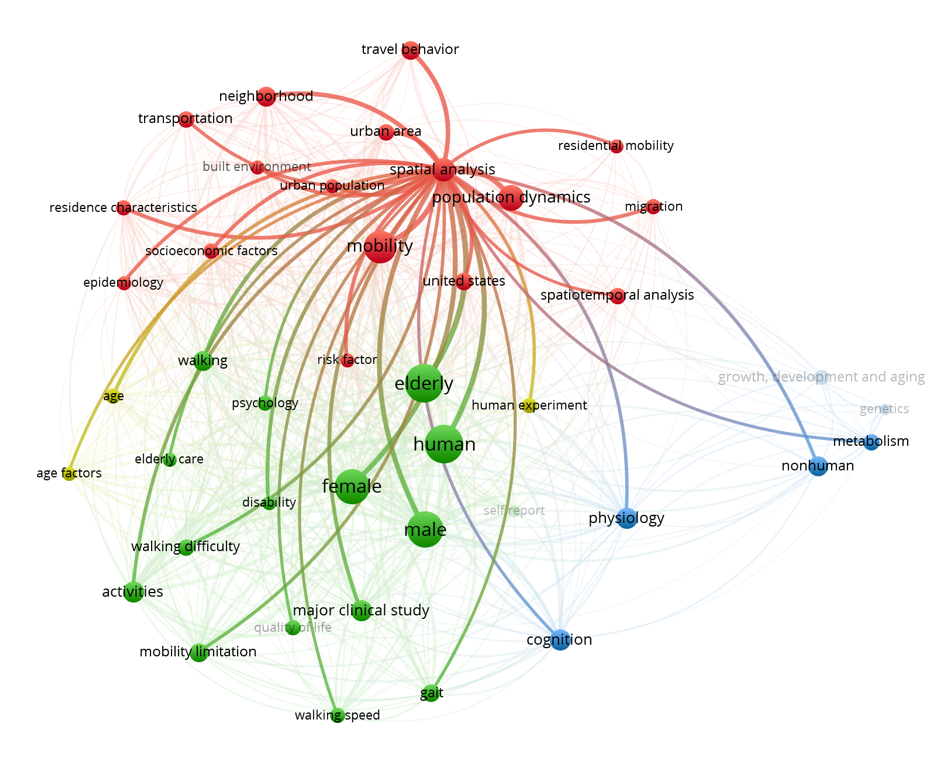 | **keywords** | **link strength** |
|  | mobility | 16 |
|  | population dynamics | 13 |
|  | neighborhood | 9 |
|  | travel behavior | 9 |
|  | urban area | 7 |
|  | walking | 5 |
|  | urban population | 4 |
|  | residence characteristics | 4 |
|  | socioeconomic factors | 4 |
|  | risk factor | 4 |

In Scopus, ‘mobility’ and ‘spatial analysis’ contain similar weights as in Web of Science. Tables A4 and A5 show the top 10 keywords linked to ‘mobility’ and ‘spatial analysis’ in Scopus that might be the determinants to the keyword search. Both terms indicate high overlapping in the list of keywords, namely in keywords with higher TLS. Differences start from fifth or sixth position, showing that ‘mobility’ is linked with ‘built environment’, ‘activities’, ‘transportation’ as well as with ‘cognition’ and ‘epidemiology’. In contrast, spatial analysis is linked with urban environment (keywords: ‘urban area’, ‘urban population’), socioeconomic factors, and risk factors.
